# Excitatory amino acid transporter 1 supports adult hippocampal neural stem cell self-renewal

**DOI:** 10.1016/j.isci.2023.107068

**Published:** 2023-06-08

**Authors:** Joshua D. Rieskamp, Ileanexis Rosado-Burgos, Jacob E. Christofi, Eliza Ansar, Dalia Einstein, Ashley E. Walters, Valentina Valentini, John P. Bruno, Elizabeth D. Kirby

**Affiliations:** 1 Department of Psychology, The Ohio State University, Columbus, OH 43210, USA; 2 Department of Neuroscience, The Ohio State University, Columbus, OH 43210, USA; 3 Chronic Brain Injury Program, The Ohio State University, Columbus, OH 43210, USA; 4 Department of Biomedical Sciences, University of Cagliari, 09124 Cagliari, Italy

**Keywords:** Neurology, Biochemistry, Molecular biology, Cell biology

## Abstract

Within the adult mammalian dentate gyrus (DG) of the hippocampus, glutamate stimulates neural stem cell (NSC) self-renewing proliferation, providing a link between adult neurogenesis and local circuit activity. Here, we show that glutamate-induced self-renewal of adult DG NSCs requires glutamate transport via excitatory amino acid transporter 1 (EAAT1) to stimulate lipogenesis. Loss of EAAT1 prevented glutamate-induced self-renewing proliferation of NSCs *in vitro* and *in vivo*, with little role evident for canonical glutamate receptors. Transcriptomics and further pathway manipulation revealed that glutamate simulation of NSCs relied on EAAT1 transport-stimulated lipogenesis. Our findings demonstrate a critical, direct role for EAAT1 in stimulating NSCs to support neurogenesis in adulthood, thereby providing insights into a non-canonical mechanism by which NSCs sense and respond to their niche.

## Highlights

• EAAT1, not glutamate receptors, stimulates adult hippocampal NSCs *in vitro*
• Loss of EAAT1 *in vivo* causes loss of NSCs and adult neurogenesis
• Glutamate transport promotes lipogenic processes in NSCs *in vitro* and *in vivo*
• Glutamate-stimulation of cultured NSCs requires fatty acid synthase activity

## Graphical abstract

**Figure:**
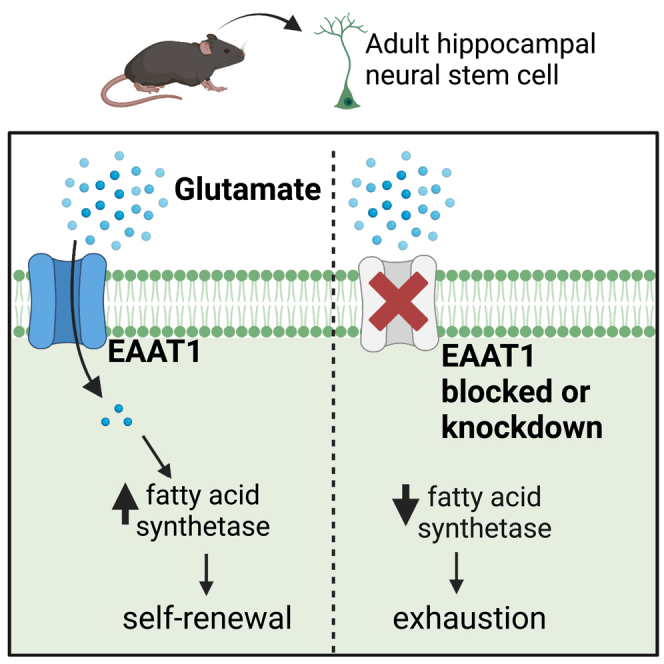

## Introduction

The dentate gyrus of the hippocampus (DG) is one of a few, isolated brain regions that can support resident neural stem cells (NSCs) that generate new neurons in adult mammals. Adult hippocampal NSCs are distinct from developmental counterparts,^1^^,^^2^^,^^3^ and their persistence throughout adulthood is conserved across most land-born mammals, likely including primates^4^^,^^5^^,^^6^^,^^7^ (but see:^8^^,^^9^). The excitatory neurotransmitter glutamate is a potent stimulator of NSC proliferation in the adult DG, a link which suggests a mechanism for tethering adult neurogenesis to circuit activity, as well as a potential mechanism for regeneration in excitotoxic conditions.^10^^,^^11^

Recent work shows that glutamate promotes asymmetric, self-renewing NSC divisions.^12^^,^^13^^,^^14^ However, the molecular mediator by which glutamate stimulates NSCs is unclear. Early studies found that NMDA receptor antagonists actually increase NSC proliferation, rather than decrease it.^15^^,^^16^^,^^17^^,^^18^ More recently, it was shown that transgenic knockout of AMPA and NMDA glutamate receptors in NSCs had no effect on their proliferation.^14^ Together, previous work suggests that the NSC response to glutamate is unlikely to rely on simple receptor stimulation.

A major component of glutamate regulation in the adult mammalian brain is the family of excitatory amino acid transporters (EAATs). EAATs are transmembrane proteins that shuttle glutamate from the extracellular to intracellular environment via ion-coupled transport. Robust EAAT expression, in particular EAAT1, is a widely used marker of adult NSC phenotype^19^^,^^20^ and *in vivo* transport-associated currents suggest these transporters are present and active in the adult DG NSC membrane.^21^^,^^22^^,^^23^ The functional role of EAATs in NSC regulation has received little attention to date. To better understand how adult NSCs interact with the glutamatergic environment of the adult mammalian DG, we set out to uncover the functional role of EAATs in these cells.

## Results

### High expression of EAAT1, but not other EAATs, is widespread in NSCs and declines rapidly with differentiation

**Figure 1:**
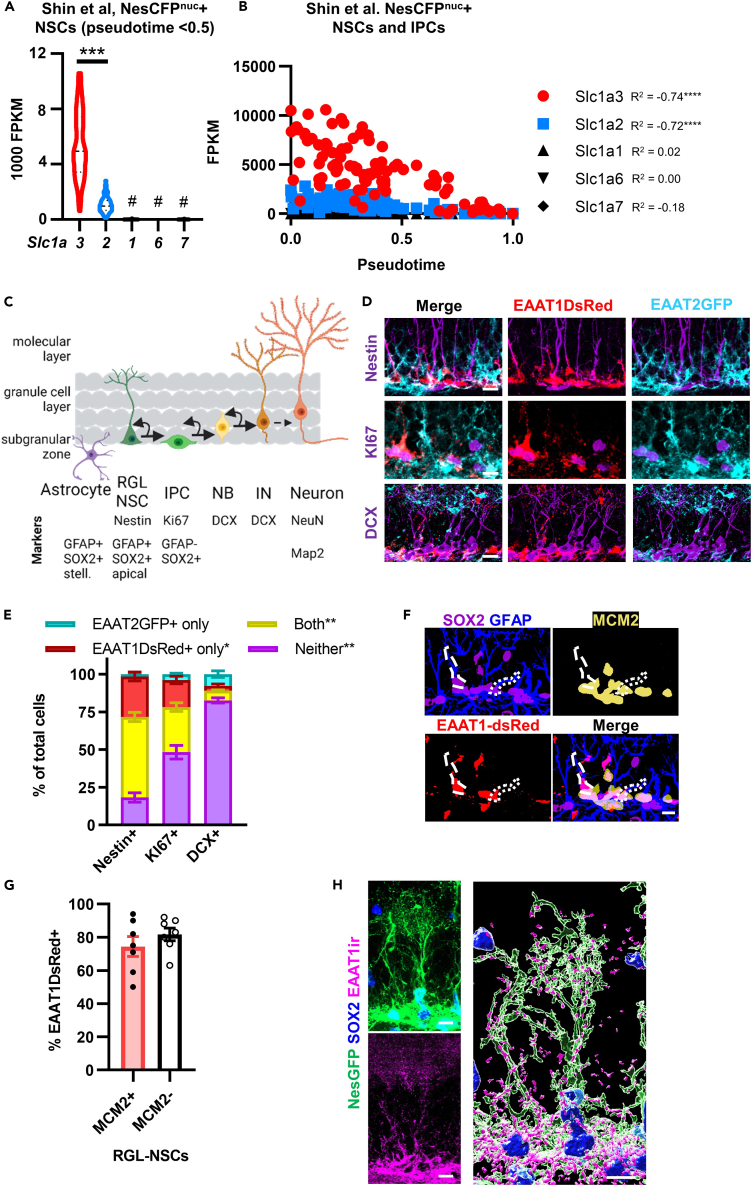
NSC express EAAT1 and to a lesser extent EAAT2 (A) scRNA-seq FPKM values for EAAT transcripts from data in Shin et al., 2015. Only cells with Pseudotime <0.5 included to reflect NSCs (as opposed to early IPCs). ∗∗∗p < 0.001, #p < 0.0001 vs. EAAT1 and 2, Dunn’s multiple comparisons. (B) FPKM of EAAT1-5 by Pseudotime from Shin et al., 2015. ∗∗∗∗p < 0.0001 Pearson correlation with Pseudotime. (C) Cells in the neurogenic cascade with immunophenotypical markers used to identify astrocytes, radial glial like NSCs (RGL NSC), intermediate progenitor cells (IPCs), neuroblasts (NB), immature neurons (IN) and mature neurons. (D) Immunolabeling for Nestin, Ki67 and DCX in DG from EAAT1DsRed+EAAT2GFP+ adult mice. Scales = 10 μm for (D, F, and H). (E) Percent of cells expressing EAAT1DsRed+EAAT2GFP+ in adult DG. ∗, ∗∗p < 0.05, < 0.01 Tukey’s post hoc test. Mean ± SEM of N = 4–5 mice. (F) Representative images for SOX2, GFAP, and MCM2 in DG from EAAT1DsRed+ adult mice. MCM2+EAAT1DsRed+ NSCs are outlined with dots, and MCM2-EAAT1DsRed+ are outlined with dashes. (G) Percent of MCM2+ or MCM2- SOX2+GFAP+ RGL-NSCs that were EAAT1DsRed+. Mean ± SEM of N = 7 mice (points shown). (H) EAAT1 immunolabeling in Nestin-GFP+ NSC, with SOX2+ nucleus. Merged z stack on left, 3D reconstruction of z stack on right. See also Figure S1.

The EAAT family of glutamate transporters has 5 primary members, EAAT1, 2, 3, 4 and 5 (gene names Slc1a3, Slc1a2, Slc1a1, Slc1a6, Slc1a7). Adult DG NSCs have been reported to express both EAAT1 and EAAT2.^21^ Using single cell RNA-seq (scRNA-seq) data from Shin et al.,^24^ we confirmed expression of EAAT1 (Slc1a3) and EAAT2 (Slc1a2) in NSCs, with EAAT2 transcript levels being ∼5.35x lower than EAAT1 transcripts (Figure 1A). Both EAAT1 and EAAT2 expression declined significantly with differentiation (Pseudotime) (Figure 1B). EAAT3 (Slc1a1), EAAT4 (Slc1a6) and EAAT5 (Slc1a7) were either completely or almost completely undetected.

To verify the extent of EAAT1 and 2 expression throughout the potentially heterogeneous population of NSCs *in vivo*, we used immunolabeling for phenotypic markers (Figure 1C) in adult EAAT1DsRed and EAAT2GFP transcriptional reporter mice^21^. 53% of Nestin+ radial glia-like NSCs were positive for both EAAT1DsRed and EAAT2GFP, with another 27% positive for EAAT1DsRed alone (Figures 1D, 1E, S1A, and S1B). Less than 2% of nestin+ NSCs were positive for EAAT2GFP alone, leaving 20% of Nestin+ NSCs negative for both transporters. Ki67+ proliferative cells in the subgranular zone, which are primarily composed of intermediate progenitor cells (IPCs), and doublecortin (DCX+) neuroblasts and immature neurons (NB/IN) each showed progressively smaller proportions of cells expressing EAATs.

To determine whether EAAT1 expression correlates with NSC activation state, we used MCM2 immunolabeling to identify cells in the cell cycle and phenotypic NSC markers SOX2 and GFAP to identify total (quiescent and active) NSCs in adult EAAT1DsRed mice (Figure 1F). The majority of both MCM2+ and MCM2-radial glia-like NSCs (74% and 81%, respectively) expressed EAAT1DsRed, with no significant difference between them (Figure 1G).

To visualize EAAT1 protein distribution throughout the full morphology of NSCs, including their apical process and bushy terminals in the molecular layer, we used EAAT1 immunolabeling in adult Nestin-GFP mice,^25^ and nestin immunolabeling in EAAT1DsRed mice. EAAT1 immunolabeling closely mimicked the distribution of EAAT1DsRed (Figure S1C), with the most intense labeling found in the cell somas in the subgranular zone. 3D reconstructions also revealed EAAT1 protein putatively co-localizing with the molecular layer bushy terminals of NSCs in both sets of mice (Figures 1H and S1C).

Together, these data suggest that EAAT1 is prominently expressed across the population of proliferating and quiescent adult DG NSCs and EAAT1 protein is distributed throughout the cell, likely including in the apical terminals where glutamatergic synapses are abundant nearby. EAAT2 is also present in NSCs but at lower levels than EAAT1. The data also suggest that expression of both transporters declines rapidly with differentiation into IPC and early neuronal phenotypes.

### Glutamate stimulates adult NSCs via EAAT-mediated transport

To begin investigating the role of EAATs in NSCs, we used cultured NSCs derived from DGs of adult male and female mice.^26^ In our previous work,^27^^,^^28^ we verified that these cultures are composed primarily of quiescent and cycling NSCs when maintained in standard conditions. Using bulk transcriptomic and proteomic data from cultured NSCs,^27^ we first confirmed that cultured NSC expression of glutamate transporter and receptor genes was similar to that of *in vivo* NSCs. As was the case *in vivo*, cultured NSCs expressed EAAT1 most abundantly, followed by EAAT2. AMPA receptor subunits, NMDA receptor subunits and metabotropic glutamate receptor transcripts were expressed at low, but detectable levels (Figures S2A–S2C). To examine expression of these transporters and receptors throughout the quiescence-activation cycle in cultured NSCs, we used single cell RNA-seq transcriptome data from our previous work.^27^ EAAT1 (*Slc1a3*) and AMPA/mGluR receptor genes were expressed most abundantly in quiescent and cycling NSCs, with downregulation generally evident in those exiting the cell cycle (presumably to differentiate) (Figure S2D). These findings generally agree with *in vivo* NSC scRNA-seq (Figure 1B), showing high *Slc1a3* in quiescent/active NSCs (Pseudotime < ∼ 0.5) and downregulation with differentiation. Together, these data show that cultured NSCs have similar expression patterns of glutamate transporters and receptors as *in vivo* NSCs.

**Figure 2:**
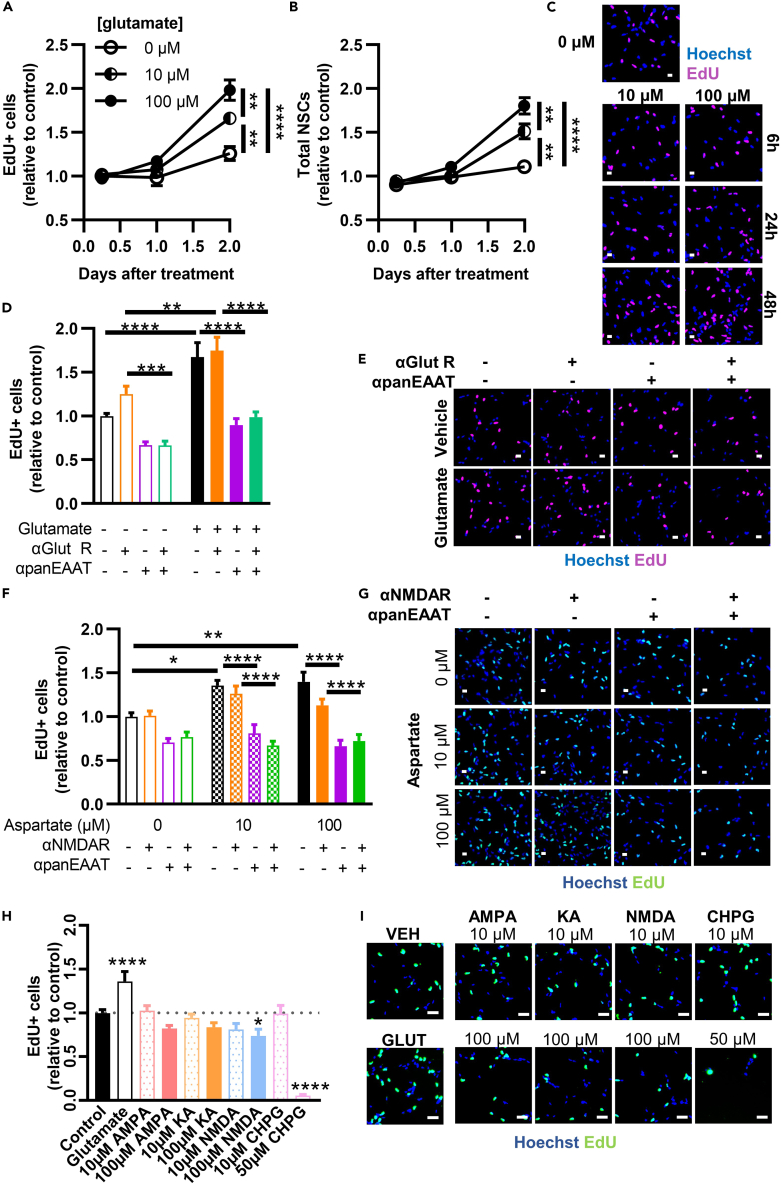
Glutamate stimulates NSC proliferation via EAATs (A) EdU+ cell counts after glutamate treatment over time. ∗∗, ∗∗∗∗p < 0.01, < 0.001 Tukey’s comparisons within timepoint. Mean ± SEM of N = 2–5 replicates/experiment, 3 independent experiments. (B) Total NSC counts after glutamate treatment over time. ∗∗, ∗∗∗∗p < 0.01, < 0.001 Tukey’s comparisons within timepoint. Mean ± SEM of N = 2–5 replicates/experiment, 3 independent experiments. (C) Representative image of EdU+ labeling in cultured NSCs after 0, 10, or 100μM glutamate treatment over time. Hoechst to label nuclei and scale = 20 μm for (C, E, G, and I). (D) EdU+ cell counts after treatment with 100 μM glutamate +/− inhibitors of glutamate receptors (αGlutR: NBQX, D-AP5, and ACDPP) or EAATs (αpanEAAT: TFB-TBOA). ∗∗,∗∗∗,∗∗∗∗p < 0.01, 0.001, 0.0001 Sidak’s multiple comparisons. Mean ± SEM of N = 5 replicates/experiment, 4 independent experiments. (E) Representative image of EdU+ labeling in cultured NSCs after glutamate +/− receptor and transporter inhibitors. (F) EdU+ cell counts after treatment with aspartate +/− inhibitor of NMDA receptor (αNMDAR) or EAATs (αpanEAAT). ∗,∗∗,∗∗∗,∗∗∗∗p < 0.05, 0.001, 0.0001 Tukey’s multiple comparisons. Mean ± SEM of N = 5 replicates/experiment, 3 independent experiments. (G) Representative image of EdU+ labeling in cultured NSCs after aspartate treatment. (H) EdU+ cell counts after treatment with glutamatergic agonists. ∗,∗∗∗∗p < 0.01, 0.0001 Dunnett’s multiple comparisons to control. Mean ± SEM of N = 6–12 replicates/experiment, 4 independent experiments. (I) Representative image of EdU+ labeling in cultured NSCs after glutamate receptor agonist treatment. See also Figure S2.

We next confirmed that glutamate stimulates proliferation of isolated NSCs using incorporation of the thymidine analog ethynyl deoxyuridine (EdU) after glutamate exposure. Glutamate increased proliferating (EdU+) and total NSC number ∼2-fold after 48h of treatment, with the effect being most notable with 100 μM glutamate (Figures 2A–2C). To determine the role of glutamate receptors versus transporters in this glutamate-induced proliferation, we treated NSCs with several pharmacologic inhibitors. Treatment with a cocktail of inhibitors to block AMPA, NMDA and mGluR5 (NBQX, D-AP5, and ACDPP, referred to together as αGlut R) had no effect on basal NSC proliferation nor their response to glutamate (Figures 2D and 2E). Treatment with the panEAAT inhibitor TFB-TBOA (αpanEAAT), in contrast, blocked glutamate-induced proliferation. Transport inhibition had this effect whether glutamate receptors were blocked or not.

EAAT1 and 2 both transport aspartate, as well as glutamate. We repeated the above experiment with aspartate and found that, similar to glutamate, aspartate stimulated NSC proliferation, and that effect was blocked by αpanEAAT inhibition (Figures 2F and 2G). Aspartate can activate NDMA receptors at higher doses. Even the NMDA receptor inhibitor (D-AP5) had no effect on NSC proliferation in any condition.

To further explore the role of glutamate receptors in NSC proliferation, we treated NSCs with glutamate receptor agonists: AMPA, KA, NMDA and CHPG (mGluR5 agonist). None of the agonists increased proliferation (Figures 2H and 2I). NMDA slightly decreased proliferation. CHPG (mGluR5) agonist caused massive cell death at its higher dose and had no effect at its lower dose. Together, these findings suggest, unexpectedly, that EAAT transporter activity alone mediates glutamate-induced NSC proliferation with no, or possibly a countering, contribution of glutamate receptors.

### Glutamate stimulation of adult NSCs relies on EAAT1 not EAAT2

**Figure 3:**
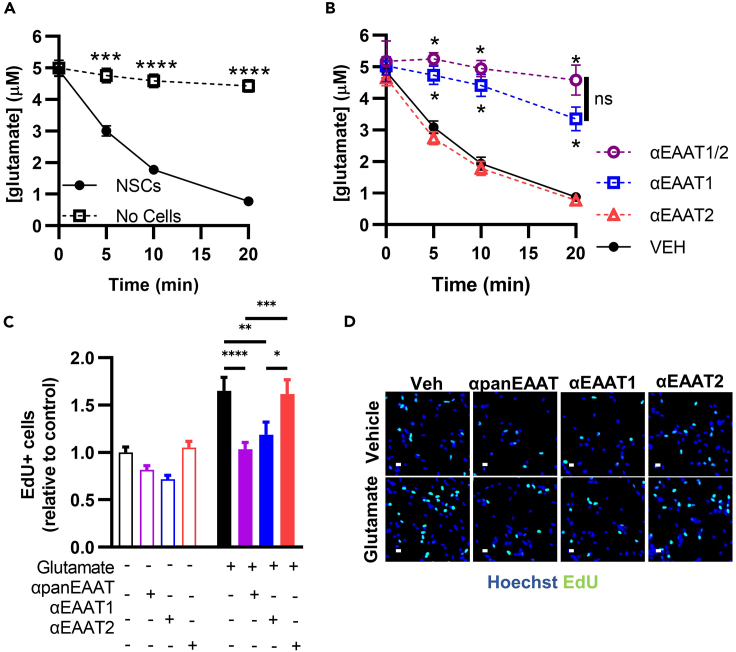
Glutamate stimulates NSC proliferation via EAAT1 (A) Glutamate in conditioned media as measured by uHPLC after a 5 μM pulse in wells with NSCs or empty wells. ∗∗∗,∗∗∗∗p < 0.001, 0.0001 vs. “No Cells” Sidak’s multiple comparisons. Mean ± SEM of N = 6 independent experiments. (B) Glutamate in NSC conditioned media as measured by uHPLC after a 5 μM pulse with pretreatment with inhibitors of EAAT1 (UCPH102), EAAT2 (WAY213613) or their combination (EAAT1/2). ∗p < 0.05 Tukey’s comparisons within timepoint. Mean ± SEM of N = 3 independent experiments. (C) EdU+ cell counts after treatment with glutamate +/− inhibitors of EAAT1 (UCPH102), EAAT2 (WAY213613), or panEAAT (TFB-TBOA). ∗,∗∗,∗∗∗,∗∗∗∗p < 0.05, 0.01, 0.001, 0.0001 Tukey’s multiple comparisons. Mean ± SEM of N = 5 replicates/experiment, 3 independent experiments. EdU+ counts expressed relative to 0 μM glutamate/vehicle within experiment. (D) Representative image of EdU+ labeling in cultured NSCs after glutamate +/− transporter inhibitors. Hoechst to label nuclei and scale = 20 μm. See also Figure S3.

To determine which EAATs might be active in NSCs, we measured glutamate clearance from NSC cultures using ultra high performance liquid chromatography with electrochemical detection (uHPLC-ECD) of conditioned media. Within 20 min, NSCs cleared 82% of a 5 μM glutamate pulse from the media (Figure 3A). Treatment with the selective EAAT1 inhibitor (UCPH101, αEAAT1) or a combination of αEAAT1 and the selective EAAT2 inhibitor (WAY213613, αEAAT2) completely prevented glutamate clearance compared to vehicle treated controls (Figure 3B). αEAAT2 alone had no effect on clearance compared to vehicle treated controls. To confirm activity of the αEAAT2 drug, we replicated the experiment with a higher dose that also inhibits EAAT1. Higher dose αEAAT2 partially prevented glutamate clearance, suggesting that the compound is active but only shows effects at doses that inhibit EAAT1 (Figure S3A). Similar results were found with an alternative EAAT2 inhibitor (DHK) (Figure S3B). Taken together, these results demonstrate functional glutamate clearance by NSCs and implicate EAAT1 as the dominant transporter.

To test the functional role of EAAT1 vs. EAAT2 in NSC proliferation, we treated cultured NSCs with αEAAT1, αEAAT2 or αpanEAAT then quantified cell proliferation using EdU labeling. Inhibiting EAAT1 alone or panEAAT inhibition prevented glutamate-induced NSC proliferation while EAAT2 inhibition alone had no effect (Figures 3C and 3D). Throughout these experiments, cells in all groups appeared healthy and showed no observable signs of apoptosis (fragmented nuclei, loss of plate adhesion). We also confirmed that neither αpanEAAT nor αEAAT1 treatment led to signs of cell death using a Sapphire700 IR dye assay (Figure S3C). Together, these data suggest that glutamate stimulates NSC proliferative expansion directly via EAAT1 activity.

### EAAT1 knockdown causes cell-autonomous loss of NSCs *in vivo*

**Figure 4:**
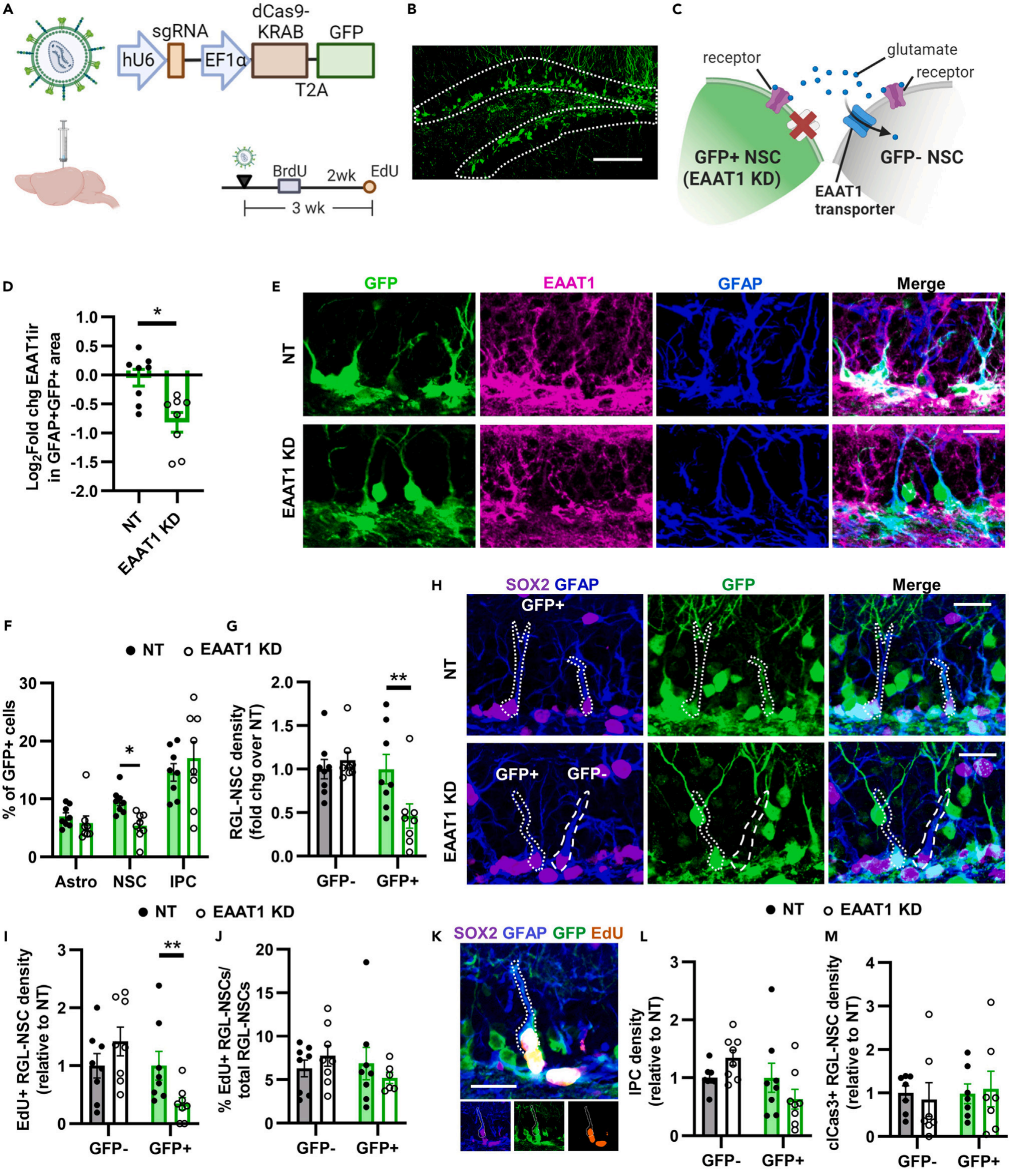
EAAT1 knockdown causes cell-autonomous loss of the NSC pool (A) CRISPRi lentiviral vector design and timeline. (B) Representative GFP+ labeling in DG at 3 weeks after infusion. Scale = 100 μm. (C) Diagram of cell-autonomous EAAT1 KD model with GFP reporter. GFP+ cells lack cell-autonomous glutamate transport mediated by EAAT1, but still contain glutamate receptors. GFP- cells have intact EAAT1-mediated glutamate transport and glutamate receptors. (D) EAAT1 immunolabeling intensity within GFP+GFAP+ area of mice infused with NT or EAAT1 KD CRISPRi lentiviral vectors in the DG. ∗p < 0.05 Mann–Whitney test. Mean ± SEM of N = 8 mice. (E) Representative image of EAAT1 labeling coupled with GFP and GFAP at 3 weeks after CRISPRi infusion. Co-labeling becomes white in merged image. Scale = 20 μm. (F) Percent of GFP+ cells expressing phenotypic markers of astrocytes (GFAP+SOX2+ stellate), NSCs (GFAP+SOX2+ radial) or IPCs (GFAP-SOX2+) 3 weeks after CRISPRi infusion. ∗p < 0.05 Sidak’s multiple comparisons. Mean ± SEM of N = 8 mice. (G) Density of GFP+ and GFP- GFAP+SOX2+ radial glia-like NSCs in NT and EAAT1 KD mice 3 weeks after infusion. Density fold change relative to NT control within GFP type. ∗∗p < 0.01 Sidak’s multiple comparisons. Mean ± SEM of N = 8 mice. (H) Representative images of GFAP, SOX2, GFP immunolabeling 3 weeks after infusion. GFP+ and GFP- NSCs are outlined. Scale = 20 μm. (I) Density of GFP+ and GFP- EdU+GFAP+SOX2+ proliferating radial glia-like NSCs in NT and EAAT1 KD mice 3 weeks after infusion. Density relative to NT control within GFP type. ∗∗p < 0.01 Sidak’s multiple comparisons. Mean ± SEM of N = 8 mice. (J) Percent of GFAP+SOX2+ proliferating radial glia-like NSCs that were EdU+ in NT and EAAT1KD mice 3 weeks after infusion. Mean ± SEM of N = 8 mice. (K) Representative image of an EdU+GFP+GFAP+SOX2+ radial glia-like NSC 3 weeks after infusion. Scale = 10 μm. (L) Density of GFP+ and GFP- GFAP-SOX2+ IPCs in NT and EAAT1 KD mice 3 weeks after infusion. Density relative to NT control within GFP type. Mean ± SEM of N = 8 mice. (M) Density of GFP+ and GFP- clCas3+GFAP+SOX2+ NSCs in NT and EAAT1 KD mice 3 weeks after infusion. See Figure S4 for representative images. Density relative to NT control within GFP type. Mean ± SEM of N = 7 mice. See also Figures S4 and S5.

To examine the role of EAAT1 in DG NSCs *in vivo*, we used CRISPR interference (CRISPRi), a transcriptional interference strategy based on the CRISPR/cas9 system. In CRISPRi, a catalytically dead Cas9 protein (dCas9) fused to a Kruppel associated box (KRAB) domain represses transcription of a gene targeted by a highly specific single guide (sg)RNA. We designed a U6→sgRNA against EAAT1 (EAAT1 KD) (and a non-target sgRNA (NT)) then inserted it into a lentiviral CRISPRi backbone^29^ with dCas9-KRAB-T2A-GFP (Figure 4A). Cultured NSCs infected with the EAAT1 KD CRISPRi vector showed a 53.1% loss of EAAT1 immunoreactivity and significant suppression of proliferation compared to NT infected cells (Figures S4A–S4D). We next stereotaxically infused the EAAT1 KD and NT CRISPRi lentiviral vectors in the DG of adult mice, where it showed strong expression as reflected by GFP immunofluorescence (Figures 4A and 4B).

In these mice, there are two possible ways that EAAT1 KD could affect NSCs: 1) cell-autonomous loss of glutamate transport in to NSCs, and/or 2) increased glutamate stimulation of receptors due to ambient, extracellular glutamate buildup after loss of EAAT1 clearance functions from NSCs and/or astrocytes. Based on our *in vitro* data, we predicted that EAAT1 supports NSC maintenance cell-autonomously. We used the GFP reporter linked to dCas9-KRAB to enable the identification of cell-autonomous versus cell-extrinsic effects of EAAT1 KD on NSC behavior. As diagrammed in Figure 4C, whereas GFP- and GFP+ NSCs can both interact with ambient glutamate via receptors, only GFP+ NSCs experience loss of cell-autonomous glutamate transport in the EAAT1 KD condition. If glutamate affects NSCs through cell-autonomous EAAT1 transport, then one would expect to observe changes in NSCs evident only in the GFP+ NSCs and not in their neighboring GFP- NSCs. On the other hand, if glutamate transport regulates NSCs via cell-extrinsic factors such as extracellular glutamate buildup and activation of glutamate receptors, one would expect changes to be evident in both GFP+ and GFP- NSCs.

1 week after CRISPRi infusion, no loss of EAAT1 protein was detected (Figures S4E–S4G). Astrocytes, IPCs and NSCs were identified using SOX2 and GFAP immunolabeling plus morphology. These cells comprised ∼34% of the GFP+ cells (Figures S4H–S4K). The remainder of the GFP+ cells were mostly (∼64%) MAP2+ mature or DCX+ immature neurons. No differences between EAAT1 KD and NT were identified in the distribution of GFP+ cells across cellular phenotypes, as expected from lack of protein level knockdown.

3 weeks after CRISPRi infusion, a 40.6% knockdown of EAAT1 immunoreactivity was detected in EAAT1 KD compared to NT treated controls (Figures 4D and 4E). In addition, EAAT2 expression was not altered by EAAT1 KD compared to NT control (Figures S5A–S5C). At this timepoint, ∼30% of GFP+ NSCs were SOX2+ astrocytes, NSCs or IPCs (Figure S5D). Among those, we found a selective reduction in the percent of GFP+ NSCs, but not in the percent of GFP+ astrocytes or IPCs (Figure 4F). To investigate the cell autonomous versus cell-extrinsic effect of EAAT1 KD as described above, we next compared GFP+ NSCs to neighboring GFP- NSCs. At 3 weeks after viral infusion, EAAT1 KD mice had 53.9% fewer GFP+GFAP+SOX2+ radial glia-like NSCs compared to NT treated controls (Figures 4G and 4H). In contrast, the neighboring GFP- NSCs in these mice showed no effect of EAAT1 KD. The number of proliferating (EdU+) GFP+GFAP+ radial glia-like NSCs showed the same pattern, with a 63.5% decrease in density of proliferating GFP+ NSCs with EAAT1 KD but no effect on neighboring GFP- proliferating NSCs compared to NT control mice (Figures 4I and 4K). No difference was found in the percent of GFP+ or GFP- NSCs that were proliferating (EdU+) between NT and EAAT1 KD mice (Figures 4J and 4K). No differences were found between NT and EAAT1 KD mice in GFP+ or GFP- GFAP-SOX2+ IPC density nor in density of DCX+ neuroblasts/immature neurons that were BrdU-labeled 2 weeks before perfusion (Figures 4L, S5E, and S5F). SOX2+GFAP+ NSCs expressing the apoptotic marker, cleaved caspase 3, were rare and no differences were found in their density between NT and EAAT1 KD mice among GFP+ or GFP- NSCs (Figures 4M and S5G). These findings suggest that loss of EAAT1 causes a cell-autonomous loss of the radial glia-like NSC pool, independent of cell death.

### Long-term EAAT1 knockdown causes cell-autonomous loss of adult neurogenesis

**Figure 5:**
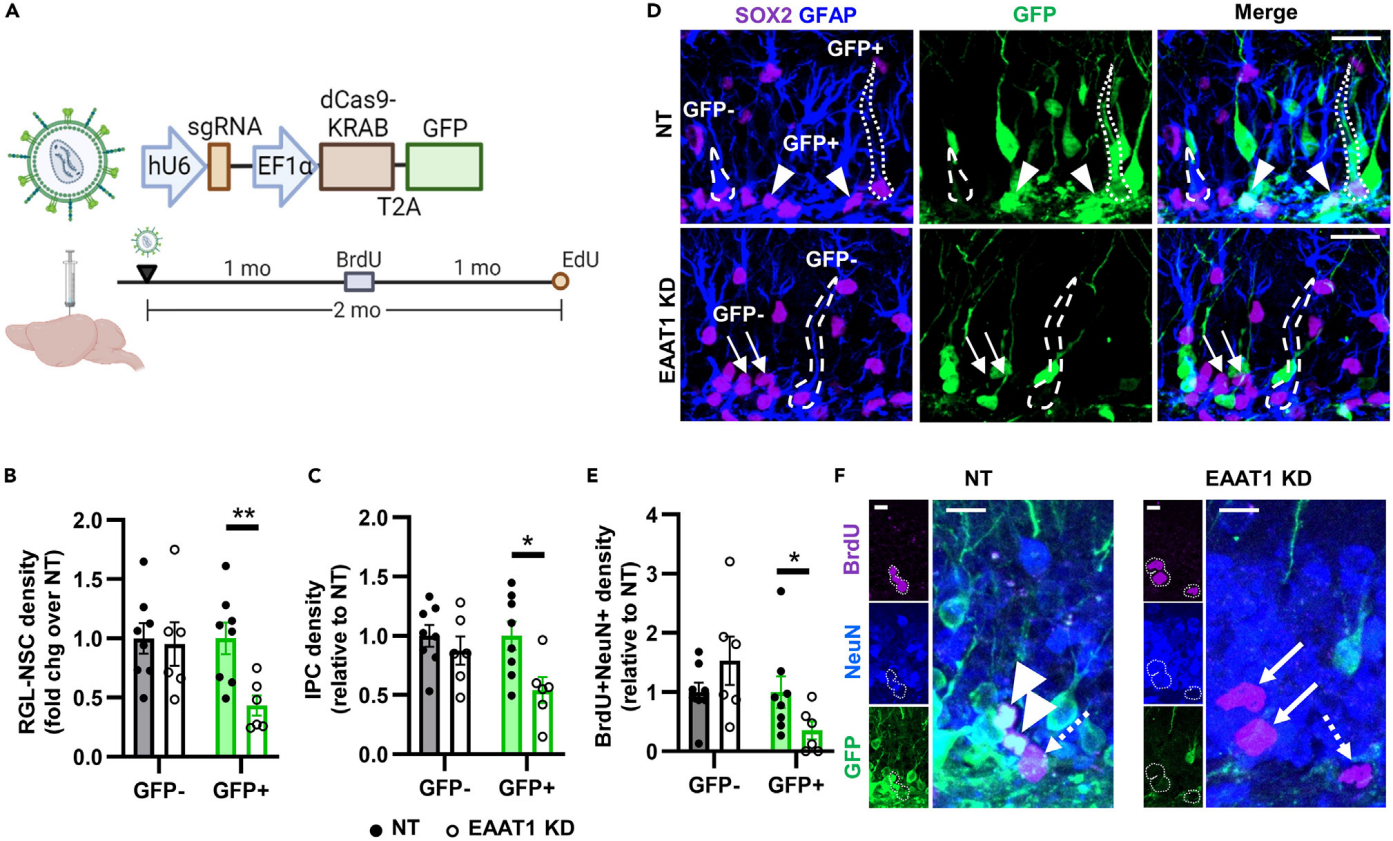
EAAT1 knockdown causes cell-autonomous loss of neuronal NSC progeny (A) Timeline for CRISPRi infusion and thymidine analog administration. (B) Density of GFP+ and GFP- GFAP+SOX2+ radial glia-like NSCs in NT and EAAT1 KD mice 2 months after infusion. Density relative to NT control within GFP type. Mean ± SEM of N = 6–8 mice. ∗∗p < 0.01 Sidak’s multiple comparisons within GFP label type. (C) Density of GFP+ and GFP- GFAP-SOX2+ IPCs in NT and EAAT1 KD mice 2 months after infusion. Density relative to NT control within GFP type. Mean ± SEM of N = 6–8 mice. ∗p < 0.05 Sidak’s multiple comparisons within GFP label type. (D) Representative images of GFAP, SOX2, GFP immunolabeling 2 months after infusion. GFP+ and GFP- NSCs are outlined. IPCs shown by arrows (GFP-) or arrowheads (GFP+). Scale = 20 μm. (E) Density of GFP+ and GFP- NeuN+BrdU+ cells in NT and EAAT1 KD mice 2 months after infusion. Density relative to NT control within GFP type. Mean ± SEM of N = 6–8 mice. ∗p < 0.05 Sidak’s multiple comparisons within GFP label type. (F) Representative images of BrdU+ cells. Arrowhead = BrdU+NeuN+GFP+, Arrow = NeuN+BrdU+, Dashed arrow = BrdU+. Scale = 10 μm.

To determine whether the loss of NSCs observed at 3 weeks after knockdown would result in loss of adult neurogenesis at longer timepoints, we perfused mice 2 months after NT or EAAT1 KD CRISPRi lentiviral infusion (Figure 5A). The cell autonomous loss of GFP+ NSCs persisted at this later timepoint. EAAT1 KD mice showed a 63.0% loss of GFP+ radial glia-like NSCs with no effect on GFP- NSCs compared to NT control mice (Figure 5B). GFP+ IPC density was also 54.3% lower in EAAT1 KD mice compared to NT control mice, whereas density of GFP- IPCs did not differ between EAAT1 KD and NT mice (Figure 5C). EAAT1 KD mice also showed a 73.6% reduction in number of GFP+NeuN+ newborn neurons labeled with BrdU 1 month before perfusion (Figures 5E and 5F). Owing to the age-related decrease in NSC proliferation, we could not identify enough EdU+ NSCs in these mice for their quantification. These data suggest that loss of EAAT1 results in loss of adult neurogenesis that is restricted to the progeny of EAAT1 KD NSCs and does not extend to neighboring NSCs and their progeny.

### Glutamate stimulates, and EAAT inhibition prevents, biosynthetic processes

**Figure 6:**
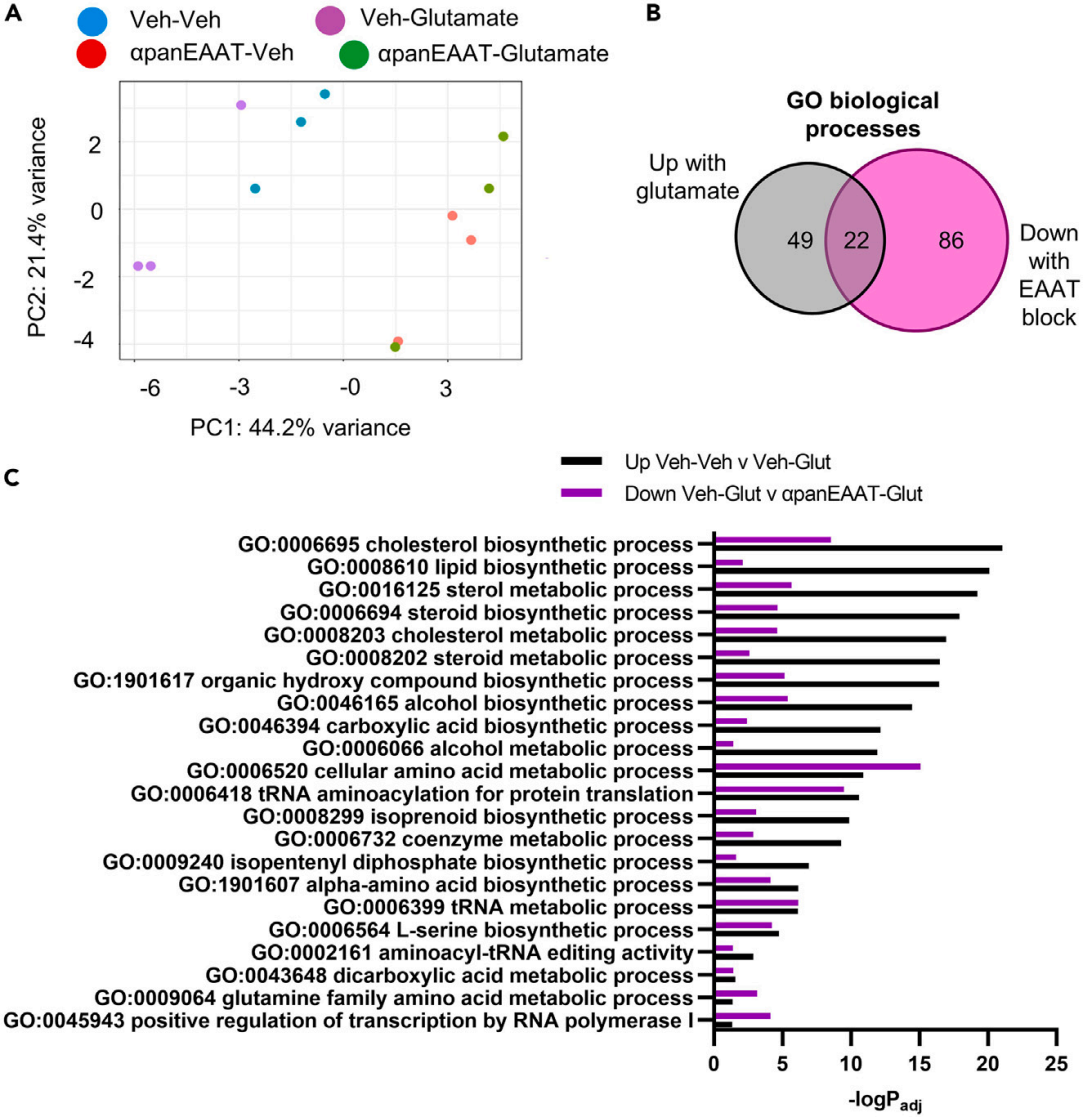
Glutamate transport is required for glutamate-induced lipogenesis and other metabolic and biosynthetic processes (A) Principal component analysis of RNA-seq samples from treated *in vitro* NSCs. (B and C)22 GO biological processes that were upregulated by glutamate and downregulated panEAAT inhibition (TFBTBOA) in RNA-seq. -logPadj shown. N = 3 replicates/group.

To gain insight into the molecular mediators of EAAT-dependent stimulation of NSC self-renewal, we performed bulk RNA-seq on RNA from cultured NSCs treated with glutamate and/or panEAAT inhibitor. Principal component analysis revealed a strong separation of groups by EAAT inhibition status (Figure 6A). In the absence of αpanEAAT, glutamate treatment caused significant upregulation of 106 transcripts and significant downregulation of 49 transcripts (Table S1). In contrast, in the presence of αpanEAAT, glutamate treatment yielded no upregulated transcripts and only 1 downregulated transcript. These data suggest robust NSC transcriptional response to glutamate that is prevented by EAAT inhibition.

To understand the functional significance of genes regulated by glutamate transport, we focused on GO biological process analysis of genes upregulated by glutamate that were also downregulated by αpanEAAT (Table S2). We found 22 overlapping GO terms which centered mostly on metabolic processes related to lipid, amino acid, and protein synthesis (Figures 6B and 6C).

### EAAT-dependent stimulation of fatty acid synthetase is essential for glutamate-induced NSC proliferation

**Figure 7:**
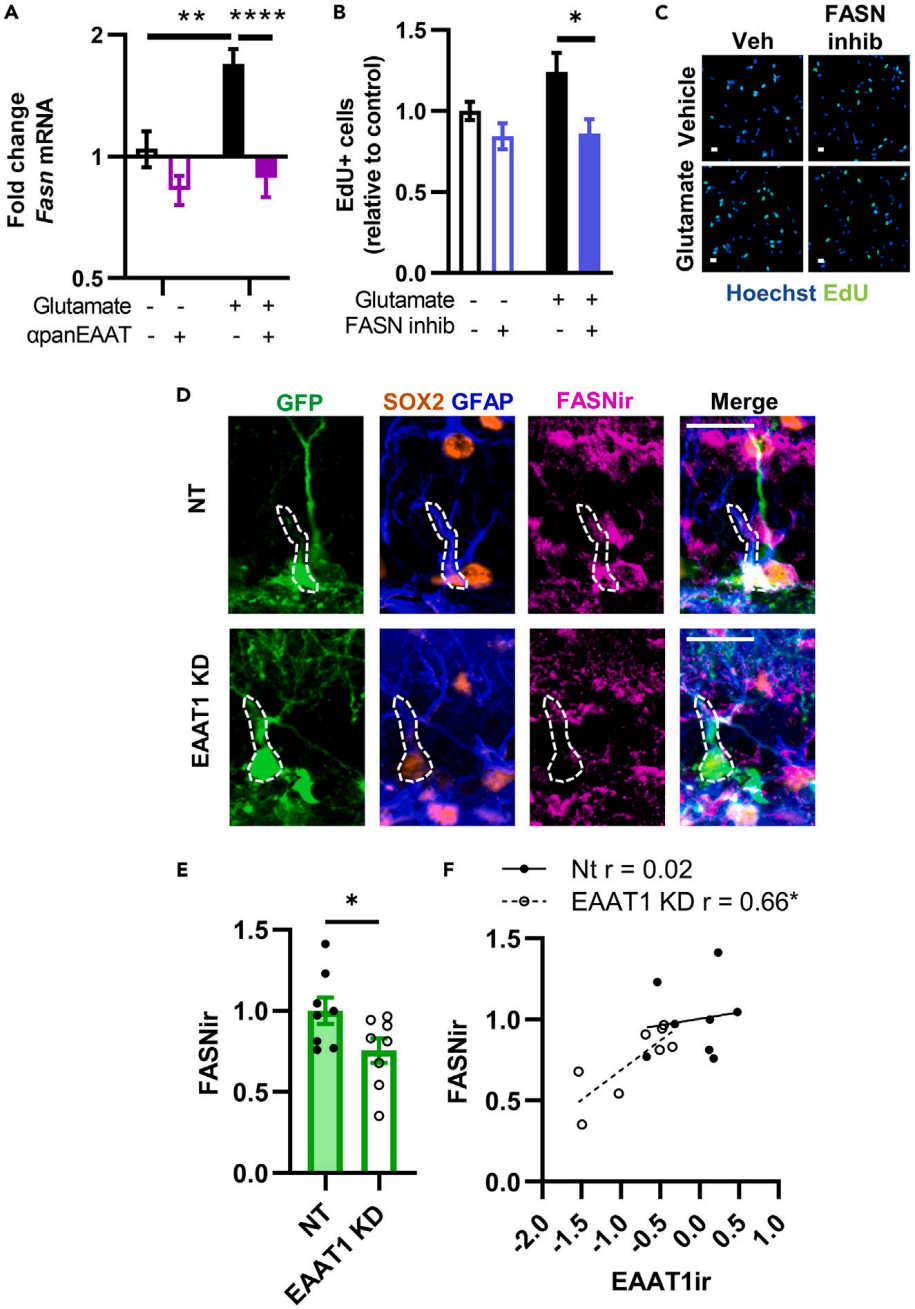
Glutamate stimulates NSC self-renewal via transport-stimulated FASN expression (A) Fasn mRNA expression in cultured NSCs as measured by real time qPCR after treatment with glutamate +/− panEAAT inhibition (TFBTBOA). ∗∗,∗∗∗∗p < 0.01, 0.0001 Sidak’s multiple comparisons. Mean ± SEM of N = 3 replicates/experiment, 3 independent experiments. (B) EdU+ cell counts *in vitro* after treatment with glutamate +/− inhibitor of FASN (orlistat). Mean ± SEM of N = 4 replicates/experiment, 4 independent experiments. ∗p < 0.05 Sidak’s multiple comparisons. (C) Representative images of EdU labeling in NSCs treated with glutamate +/− FASN inhibitor orlistat. Hoechst as nuclear marker. Scale = 10 μm. (D) Representative images of GFAP, SOX2 and FASN immunolabeling in mice treated with CRISPRi NT or EAAT1 KD lentiviral vectors 3 weeks after viral infusion. Example radial glia-like NSCs are outlined. Scale = 20 μm. (E) FASN immunolabeling (FASNir) intensity within GFP+GFAP+SOX2+ radial glia-like NSCs of mice infused with NT or EAAT1 KD CRISPRi lentiviral vectors in the DG 3 weeks before perfusion. Mean ± SEM of N = 8 individual mice (points shown). ∗p < 0.05 t-test. (F) Correlation of FASNir with EAAT1ir in GFP+GFAP+SOX2+ radial glia-like NSCs of mice infused with NT or EAAT1 KD CRISPRi lentiviral vectors in the DG 3 weeks before perfusion. Mean ± SEM of N = 8 individual mice (points shown). ∗p < 0.05 Spearman correlation.

Particularly notable among the cellular processes implicated in EAAT-dependent regulation of NSC gene expression above was lipid biosynthetic process. Lipogenesis is essential for NSC self-renewal, with a particularly critical role for fatty acid synthetase (FASN).^30^^,^^31^^,^^32^ In our RNA-seq data, glutamate treatment induced upregulation of *Fasn*, and that effect was blocked by EAAT inhibition (Tables S1 and S3). Independent replication using qPCR confirmed that glutamate stimulated *Fasn* expression in an EAAT-dependent manner (Figure 7A). To test the functional role of *Fasn* in glutamate-stimulated NSC proliferation, we treated cultured NSCs with the FASN inhibitor, orlistat. FASN inhibition prevented glutamate-induced NSC proliferation (Figures 7B and 7C). These findings suggest that glutamate transport by EAAT promotes NSC proliferation via stimulation of FASN. To determine whether these findings might extend *in vivo*, we used immunolabeling for FASN in mice infused 3 weeks prior with NT and EAAT1 KD CRISPRi lentiviral vectors. We found that FASN immunoreactivity in GFP+ NSCs was reduced by ∼25% in EAAT1 KD mice compared to NT mice (Figures 7D and 7E). We also found that FASN immunoreactivity correlated significantly with EAAT1 immunoreactivity in EAAT1 KD mice, but not in NT mice (Figure 7F). Together, these findings suggest that glutamate transport stimulation of NSCs relies on FASN expression.

## Discussion

Our findings suggest a functional role for EAAT1 in supporting adult hippocampal neurogenesis via cell autonomous NSC pool maintenance. Our findings stand in contrast to a canonical glutamate receptor-dependent mechanism reported in most other cell types and suggest that EAAT1 expressed by NSCs directly supports self-renewing proliferation via transport-dependent stimulation of lipogenesis.

We found that loss of EAAT1 led to cell-autonomous loss of radial glial-like NSCs and their progeny. The majority of adult NSCs are quiescent and the maintenance of that quiescence is essential for sustaining adult neurogenesis throughout the lifespan.^33^^,^^34^ Ectopic activation from quiescence can result in exhaustion of the NSC pool, as can prevention of reentry into quiescence after activation.^35^^,^^36^^,^^37^ The decrease in the NSC pool that we observed after EAAT1 KD could suggest disrupted quiescence through either of these mechanisms. However, a disruption would also result in a shift in the percent of NSCs that were quiescent versus active. We did not observe any change in the percent of active (proliferating) NSCs after EAAT1 knockdown. Our findings therefore suggest that quiescence itself is not regulated by EAAT1.

After activation, NSCs can undergo one of several possible modes of division (Figure S6). Symmetric, expansive divisions grow the NSC pool by generating two NSCs (and no new IPCs). Asymmetric, self-renewing divisions maintain the NSC pool by regenerating one NSC and fueling generation of new neurons by generating an IPC. Exhaustive, symmetric divisions deplete the NSC pool by generating two IPCs, leading to a transient surge in new neuron generation followed by exhaustion. Exhaustive asymmetric divisions have also been reported in adult mice,^38^ in which division results in an IPC and a terminally differentiated astrocyte. Balance of these division modes is necessary to both maintain the NSC pool and maintain basal levels of neurogenesis (for reviews see:^33^^,^^39^).

Our data suggest a cell autonomous shift in NSC division mode away from self-renewing divisions toward more exhaustive divisions after EAAT1 loss. We base this conclusion on our observation of loss of total and proliferating NSCs that was restricted to NSCs expressing EAAT1 KD machinery and was not accompanied by any observed increases in expression of cell death markers *in vivo*. Our *in vitro* findings similarly supported this conclusion, showing that glutamate stimulates proliferative expansion of NSC cultures in an EAAT1-dependent manner.

Perplexingly, one would usually expect a shift toward exhaustive divisions *in vivo* to be accompanied by either a transient increase in the number of IPCs and neurons or an increase in astrocyte generation. We did not see evidence of increases in IPC or astrocytic progeny of NSCs that lost EAAT1 *in vivo*. Rather, we observed a slow decline in IPC and new neuron generation after loss of the NSC pool. In some ways, our data resemble the common phenotype of aging: a slow loss of NSCs and neurogenesis. The cellular mechanisms of age-related loss of neurogenic capacity have generated great debate over the past 10–15 years.^38^^,^^39^^,^^40^ Recent evidence suggests multiple, coincident changes in NSCs and their progeny that all contribute to age-related decline in neurogenesis.^34^^,^^41^^,^^42^^,^^43^^,^^44^ Most relevant to our data, aging appears to be accompanied at least in part by exhaustive, symmetric NSC divisions, coupled with reduced clonal expansion of IPCs. A likely explanation for our data, then, is that EAAT1 loss accelerates an aging-like phenotype of exhaustive division of NSCs coupled with reduced proliferative expansion of IPCs. More research is needed to confirm these possibilities and to determine if EAAT1 is necessary for IPC expansion directly or as a downstream consequence of deriving from an NSC lacking EAAT1.

Expression of glutamate transporter proteins, particularly EAAT1, is widespread in the radial glia NSCs of the embryonic forebrain.^45^ One study in early postnatal DG NSCs concluded that EAATs indirectly suppress proliferation by limiting the activation of mGluRs.^46^ This apparent reversal of the roles of EAAT1 and mGluR5 in early postnatal hippocampus versus what we observed in adult DG NSCs is paralleled by downregulated expression of mGluR5 and upregulated expression of EAAT1 in DG NSCs during the transition to adulthood.^47^ This transition could contribute to the shift in cellular behavior of DG NSCs from rapidly proliferating and depleting early in life to more maintenance-focused later in life.^34^ Comparative studies of EAAT function in NSCs across different stages of development could shed further light on this hypothesis.

*In vitro*, we repeatedly noticed a suppression of NSC proliferation with EAAT inhibition (pharmacologic or genetic) in no-glutamate conditions. This pattern did not always reach statistical significance but was repeated across many experiments. Although standard NSC culture media has no added glutamate, it does have ample glutamine. Astrocytes, which also express EAATs, have been shown synthesize glutamate from other precursors, such as glutamine, and release it in culture.^48^ It therefore seems possible that NSCs could synthesize and release small amounts of glutamate that they then recycle back through EAAT1, making EAAT1 active even without added exogenous glutamate. Future studies could address whether a low basal EAAT1 activity *in vitro* could be critical to NSC long-term proliferation in typical culture conditions. This mechanism would most likely be a feature of cultured cells, though, as glutamate from local circuit activity is likely a much more abundant source of glutamate *in vivo*.

Cell metabolic states are becoming increasingly recognized as active players in NSC self-renewal and fate decisions, both as generators of key cellular substrates for growth and as independent initiators of gene expression.^49^^,^^50^^,^^51^ Several previous studies demonstrate a particularly critical role for fatty acid synthesis in DG NSC self-renewal.^30^^,^^31^^,^^32^ In agreement with this previous data, we found that FASN activity was required for glutamate-induced NSC proliferation *in vitro* and that FASN expression was suppressed after EAAT1 loss in NSCs *in vivo*. Cell metabolism, and lipogenesis in particular, therefore represent a potential convergence point for the glutamate-stimulated, EAAT-dependent processes that we observed in adult DG NSCs. A remaining open question, however, is how transport of glutamate stimulates lipogenesis. Glutamate transport both provides glutamate as a substrate to the intracellular environment and a net inward positive current because of ionic co-transport.^52^ Either or both of these stimuli could underpin the connection between glutamate transport and lipogenesis. Future research will be needed to address these questions.

NSCs may derive glutamatergic input from multiple sources *in vivo*. NSC apical processes wrap around putatively glutamatergic synaptic terminals in the molecular layer of the DG.^53^ Inputs here from hilar mossy cells^14^ and hypothalamus^13^ both stimulate NSC self-renewal, as does release from local astrocytes.^12^ With multiple sources of glutamate, it is tempting to speculate that changes in hippocampal glutamate signaling that occur during normal development, neurodegenerative pathology, and plasticity-inducing stimuli^54^^,^^55^^,^^56^^,^^57^ could be transduced by EAAT1 into metabolic states that regulate the population maintenance of NSCs. Future studies could examine whether changes in EAAT expression or function modulate the response of NSCs to these physiological states.

In summary, we show that glutamate transport via EAAT1 cell-autonomously supports DG NSC self-renewal in adult mice. These findings help resolve some discrepancy in the field about the role of glutamate receptors versus glutamate itself in NSC maintenance. They also raise additional questions about the interplay between glutamate signaling and NSC behavior in contexts such as biological aging, neurological disorders, and plasticity-inducing stimuli like exercise and enriched environment.^10^^,^^58^^,^^59^

### Limitations of the study

In our *in vitro* studies, the glutamate concentration at the end of 48h of glutamate treatment is unclear. uHPLC measurement requires use of aCSF as media and therefore a short assay time because NSCs cannot survive 48h in this media. It therefore remains unknown how glutamate concentration changed over the course of days in these experiments. In addition, our *in vitro* data show that FASN is required for glutamate stimulation of NSCs and our *in vivo* data present correlative evidence that glutamate transport stimulates NSCs via FASN lipogenesis, but causative studies are need to further confirm the role of FASN *in vivo*. The presented studies only examine NSCs in the adult DG of mice. The NSCs in the adult subventricular zone neurogenic niche also express EAAT1,^60^ and the role of EAAT1 in this niche remains an open question, to the best of our knowledge. Whether these results extend to other species, including primates, is also not yet clear. However, a recent scRNA-seq profiling of adult macaque hippocampal NSCs show high levels of EAAT1 and EAAT2 expression,^61^ suggesting the possibility that NSCs in adult primate hippocampus could rely on EAAT-mediated glutamate transport. We also do not resolve which glutamatergic inputs impact NSCs directly via EAAT1, nor how EAATs come in to play in pathological conditions characterized by excess glutamate transmission. Our work only begins the uncovering of the intracellular signaling between glutamate transport and stimulation NSC self-renewal. These steps will require more detailed investigation in the future.

## STAR★Methods

### Key resources table

**Table:** 

REAGENT or RESOURCE	SOURCE	IDENTIFIER
**Antibodies**		

Rabbit anti-mCherry/dsRed (1:500 IF)	Abcam	Cat#ab167453; RRID:AB_2571870
Mouse anti-mCherry/dsRed (1:500 IF)	Novus Biologicals	Cat#NBP1-96752; RRID:AB_11034849
Goat anti-GFP (1:1000 IF)	Abcam	Cat#ab6673; RRID:AB_305643
Mouse anti-Nestin (1:100 IF)	Millipore-Sigma	Cat#MAB353; RRID:AB_94911
Rat anti-Ki67 (1:500 IF)	Invitrogen	Cat#14-5698-82; RRID:AB_10854564
Rabbit anti-DCX (1:500 IF)	Cell Signaling	Cat#4604; RRID:AB_561007
Rabbit anti-EAAT1 (1:250 IF)	Abcam	Cat#ab416; RRID:AB_304334
Guinea Pig anti-EAAT2 (1:1000 IF)	Sigma	Cat#ab1783; RRID:AB_90949
Rabbit anti-GFAP (1:1000 IF)	Dako	Cat#Z0334; RRID:AB_10013382
Mouse anti-GFAP (1:1000 IF)	Millipore	Cat#MAB360; RRID:AB_11212597
Rat anti-SOX2 (1:1000 IF)	eBioscience	Cat#14-9811; RRID:AB_11219070
Rat anti-BrdU (1:500 IF)	BioRad	Cat#OT0030; RRID:AB_305426
Rabbit anti-FASN (1:500 IF)	Abcam	Cat#ab128870; RRID:AB_11143436
Mouse anti-NeuN (1:500 IF)	Millipore	Cat#MAB377; RRID:AB_2298772
Rabbit anti-MAP2 (1:250 IF)	Cell Signaling	Cat#8707; RRID:AB_2722660
Rabbit anti-MCM2 (1:500)	Cell Signaling	Cat#4007; RRID:AB_2142134
Rabbit anti-clCas3 (1:400 IF)	Cell Signaling	Cat#9661; RRID:AB_2341188
AlexaFluor 350 anti-rabbit	Invitrogen/Fisher	Cat#A10039; RRID:AB_2534015
AlexaFluor 488 anti-goat	Invitrogen/Fisher	Cat#A11055; RRID:AB_2534102
AlexaFluor 555 anti-rabbit	Invitrogen/Fisher	Cat#A31572; RRID:AB_162543
AlexaFluor 647 anti-mouse	Invitrogen/Fisher	Cat# A31571; RRID:AB_162542
AlexaFluor 647 anti-rat	Jackson/Fisher	Cat#NC0692397; RRID:AB_2340694
AlexaFluor 647 anti-rabbit	Invitrogen/Fisher	Cat#A31573; RRID:AB_2536183
AlexaFluor 555 anti-mouse	Invitrogen/Fisher	Cat# A31570; RRID:AB_2536180
AlexaFluor 594 anti-rat	Invitrogen/Fisher	Cat# 50-194-3617; RRID:AB_2340692

**Bacterial and virus strains**		

EF1a-dCas9-KRAB-GFP backbone	This paper	Addgene Plasmid#194281
U6-Slc1a3 sgRNA; EF1a-dCas9-KRAB-GFP	This paper	Addgene Plasmid#194283
U6-NT sgRNA; EF1a-dCas9-KRAB-GFP	This paper	Addgene Plasmid#194284
pLV hU6-sgRNA hUbC-dCas9-KRAB-T2a-GFP	Charles Gersbach^29^	Addgene Plasmid#71237; RRID:Addgene_71237
VSV-G pseudotyped second generation lentivirus	Vigene Biosciences	N/A

**Chemicals, peptides, and recombinant proteins**		

L-glutamic acid (glutamate) (5, 10 or 100 μM)	Millipore-Sigma	Cat#G8415
L-aspartic acid (aspartate) (10 or 100 μM)	Tocris	Cat#214
TFB-TBOA (1 μM)	Tocris	Cat#2532
UCPH-102 (10 μM)	Abcam	Cat#ab146404
Dihydrokainic acid (DHK) (100 μM)	Tocris	Cat#0111
WAY 213613 (1 or 10 μM)	Tocris	Cat#2652
NBQX disodium salt (10 μM)	Tocris	Cat#1044
D-AP5 (100 μM)	Tocris	Cat#0106
ACDPP hydrochloride (1 μM)	Tocris	Cat#2254
(S)-AMPA (10 or 100 μM)	Tocris	Cat#0254
Kainic acid (10 or 100 μM)	Millipore-Sigma	Cat#420318
NMDA (10 or 100 μM)	Tocris	Cat#0114
CHPG sodium salt (10 or 50 μM)	Tocris	Cat#3695
SEA 0400 (1 μM)	Tocris	Cat#6164
YM 244769 (2 μM)	Tocris	Cat#4544
Dantrolene, sodium salt (10 μM)	Tocris	Cat#0507
EGCG (50 μM)	Tocris	Cat#4524
Hoechst 33342	Fisher	Cat#H3570
Azide-biotin	Click Chemistry Tools	Cat#1265-5
AZDye 488 Azide	Click Chemistry Tools	Cat#1275-1
AZDye 647 Azide	Click Chemistry Tools	Cat#1299-1
Streptavidin Alexa Fluor 555	Invitrogen	Cat# S32355
ProLong Gold Antifading Reagent	ThermoFisher	Cat#P36934
Neurobasal A medium	Fisher	Cat#10-888-022
Percoll	VWR	Cat#89428-522
Animal-Free Recombinant Human EGF, PeproTech (AF10015-500UG) (100-18B-250UG)	VWR	Cat#:10781-694
PeproTech Recombinant Human FGF-Basic (154A.A)	VWR	Cat#10771-938
Gibco™ GlutaMAX™ Supplement	Fisher	Cat#35050-061
Laminin	VWR	Cat#47743-734
Poly-D-Lysine	Sigma	Cat#P0296
TRITON™ X-100	Thermo Scientific Chemicals	Cat#: 21568
Papain from *Carica papaya*	Sigma	Cat#10108014001 Roche
Dispase	VWR	Cat#MSPP-7913
DNase I	Fisher	Cat#NC9007308
Gibco B-27 Supplement (50X), minus vitamin A	Fisher	Cat#12-587-010
5-Ethynyl-2′-deoxyuridine	Click Chemistry Tools	Cat#1149
Accutase	Stem Cell Technologies	Cat#07920
5-Bromo-2′-deoxyuridine (BrdU)	Sigma	Cat#B5002
5-ethynyl-2′deoxyuridine (EdU)	Fisher	Cat#NC1296287

**Critical commercial assays**		

Click-&-Go® Plus EdU 488	Click Chemistry Tools	Cat#1350
Click-&-Go® EdU 647	Click Chemistry Tools	Cat#1329
NucleoSpin RNA Plus XS kit	Takara	Cat#740990.10
Qubit RNA HS assay kit	Fisher	Cat#Q32852
Clontech SMART-Seq HT kir	Takara	Cat#634455

**Deposited data**		

Raw and analyzed RNAseq data	This paper	GSE217059

**Experimental models: Cell lines**		

Cultured adult DG Neural Stem Cells	Derived as per Babu et al., 2011^26^	

**Experimental models: Organisms/strains**		

C57BL/6J mice	Jackson Labs	Cat#000664; RRID:IMSR_JAX:000664
GLAST/EAAT1-DsRed	Regan et al., 2007^21^	N/A
GLT-1/EAAT2-GFP	Regan et al., 2007^21^	N/A
NestinGFP	Jackson Labs Mignone et al., 2004^25^	Cat#033927; RRID:IMSR_JAX:033927

**Oligonucleotides**		

5′ GGACG(N)20 3′	Integrated DNA Technologies	N/A
5′ AAAC(N′)20C 3′	Integrated DNA Technologies	N/A

**Software and algorithms**		

ImageJ	schneider	https://imagej.nih.gov/ij/ RRID:SCR_003070
Zen Blue	Zeiss	https://www.zeiss.com/microscopy/us/products/microscope-software/zen.html RRID:SCR_013672
Prism	GraphPad	https://www.graphpad.com/scientific-software/prism/ RRID:SCR_002798
Broad Institute GPP portal: CRISPick	MIT Broad Institute^62^^,^^63^	https://portals.broadinstitute.org/gppx/crispick/public
DESeq R package	Bioconductor^64^	https://bioconductor.org/packages/release/bioc/html/DESeq2.html
Cytoscape 3.9.1	Shannon et al., 2003^65^	https://cytoscape.org/ RRID:SCR_003032
ClueGO	Bindea et al., 2009^66^	https://apps.cytoscape.org/apps/cluego RRID:SCR_005748
Biorender		https://www.biorender.com RRID:SCR_018361
Adapter Removal v2.2.0	Schubert et al., 2016^67^	https://adapterremoval.readthedocs.io/en/stable/ RRID:SCR_011834
HISAT2 v2.0.6	Kim et al., 2015^68^	http://daehwankimlab.github.io/hisat2/ RRID:SCR_015530
Subread package v1.5.1	Liao et al., 2013,^69^ Liao et al., 2019^70^	https://bioconductor.org/packages/release/bioc/html/Rsubread.html RRID:SCR_009803

### Resource availability

#### Lead contact

Further information and requests for resources and reagents should be directed to and will be fulfilled by the lead contact, Elizabeth Kirby (kirby.224@osu.edu).

#### Materials availability

Plasmids generated in this study have been deposited to Addgene, U6-Slc1a3 sgRNA; EF1a-dCas9-KRAB-GFP #194283, U6-NT sgRNA; EF1a-dCas9-KRAB-GFP #194284; pLV hU6-sgRNA hUbC-dCas9-KRAB-T2a-GFP #71237.

### Experimental model and study participant details

#### Animals

All experimental protocols were performed in accordance with institutional guidelines approved by the Ohio State University Institutional Animal Care and Use Committee and with recommendations of the National Institutes of Health Guide for the Care and Use of Laboratory Animals. For lentiviral infusion, seven week old C57BL/6J mice were obtained from The Jackson Laboratory were allowed to acclimate for 1 week prior to surgery, housed 5/cage. Mice were randomly assigned to viral timepoints by whole cage. GLAST/EAAT1DsRed and GLT-1/EAAT2-GFP mice^21^ were obtained as a kind gift from Dr. Jeffrey Rothstein and crossed to obtain offspring used in experiments. NestinGFP mice^25^ were obtained from The Jackson Laboratory and maintained in house, crossed to C57BL/6J. From the time of weaning, mice were group housed (2–5 per cage) in ventilated cages with *ad libitum* access to food and water and maintained on a 12-hour light cycle. Reporter mice aged 8–12 weeks were used for tissue staining. Both male and female mice were used in all experiments. No mouse experienced any manipulation other than standard husbandry and those described here.

#### DG NSC culture

NSCs were derived from the DG of 6 week old C57BL/6J mice based on a published protocol.^26^ Briefly, mice were euthanized by CO_2_ inhalation and brains were rapidly dissected. Bilateral DG were isolated from each brain in ice-cold Neurobasal A media and minced with a scalpel blade prior to 20 min incubation in papain/dispase/DNase I at 37°C. The mixture was then triturated with a P1000 pipette and centrifuged for 2 min at 800 x g. The pellet was resuspended in warm Neurobasal A and mixed with a 22% Percoll suspension prior to centrifugation for 15 min at 450 x g. The resulting pellet was washed several times in warm Neurobasal A prior to resuspension in complete growth media containing 1% B27 supplement, 0.5% GlutaMax, and 20 ng/mL each of epidermal growth factor (EGF) and basic fibroblast growth factor (FGF2). NSCs were maintained in adherent monolayer culture on poly-D-lysine and laminin coated plates. All experiments used cells between passages 4 and 20 and were replicated in two separate cell lines, one derived from 4 pooled male DG and the other from 4 pooled female DG. Both lines were mycoplasma tested and confirmed to produce neurons and glia upon growth factor withdrawal as previously described.^27^ In our previous work^28^ using scRNAseq, we found that ∼50% of the cells in the cultures were NSCs in the cell cycle. 17.5% showed profiles of quiescent NSCs. 14% showed transcriptional profile of injured or dying cells. Only 3% showed genes indicative of further differentiation. Cell cycle regression analysis collapsed several subpopulations within the cell cycle but did not lead to the identification of any notable IPC-like cluster.

### Method details

#### Perfusion and tissue harvest

Mice were injected with a ketamine/xylazine mixture (87.5 mg/kg, 12.5 mg/kg, i.p.) and transcardially perfused with ice-cold PBS. Brains were collected and fixed for 24h in 4% paraformaldehyde in 0.1 M phosphate buffer prior to equilibration for at least 2 days in 30% sucrose in PBS, both at 4°C. They were then sliced on a freezing microtome (Leica) in a 1 in 12 series of 40 μm slices and stored in cryoprotectant at −20°C.

#### Immunofluorescence and thymidine analog detection in brain tissue

Sections were rinsed 3x with PBS and then incubated in 1% normal donkey serum (Jackson ImmunoResearch), 0.3% Triton X-100 (blocking buffer) for 1 hour before incubating in primary antibodies (key resources table) diluted in blocking buffer overnight at 4°C with rocking. On the second day, sections were rinsed 3x with PBS and incubated in fluorophore-conjugated secondary antibodies (key resources table) diluted 1:500 in blocking buffer for 2 hours before incubation for 10 min in Hoechst 33342 (1:2000 in PBS), except for sections that were instead incubated with AlexaFluor 350 secondary antibodies, and a final 3x PBS rinse. They were then mounted on charged glass slides and coverslipped with ProLong Gold fluorescence mounting media.

For EAAT1 immunolabeling, the above procedure was followed except that tissue was pretreated with ice cold methanol for 10 min for antigen retrieval and incubated in primary antibody at 37°C overnight with shaking to increase antibody penetration.

For nestin immunolabeling, antigen retrieval was performed using citrate buffer, pH = 6.0, 10 min at 95°C prior to blocking and primary antibody incubation.

For detection of thymidine analogs, the staining process above was used with the following modifications. Tissue sections were incubated for 30 minutes in 2 N HCl at 37°C prior to incubation in anti-BrdU primary antibodies. For detection of EdU, adherent cells or tissue sections were permeabilized for 20 minutes in 0.5% Triton X-100 and incubated in the dark for 30 minutes with copper-dependent click reaction mixture (Click-&-Go® Plus EdU Cell Proliferation Assays) to label EdU with Azide-biotin for subsequent Streptavidin-Alexa Fluor 555 conjugation. Primary antibody dilutions are listed in the key resources table.

#### Image acquisition and analysis

All imaging was performed on a Zeiss Axio Observer Z.1 with apotome digital imaging system and Axiocam 506 monochrome camera (Zeiss). Images of cultured NSCs captured using Zen Blue software (Zeiss) were converted to Tag Image File Format (Tiff) for automated cell counting using ImageJ. Tissue sections were imaged using a 20x objective as z-stacks with 20× 1 μm steps. Images were analyzed using Zen Blue software (Zeiss). RGLs and IPCs, EdU+ cells, DCX+BrdU+ cells and NeuN+BrdU+ cells were counted and/or phenotyped by colocalization with cell type identity markers within the SGZ (or SGZ plus GCL for BrdU+ cells). For density measures, the number of cells was divided by the area (in μm^2^) sampled for counting. Density was then normalized to NT levels within GFP+ or GFP- to allow visualization of GFP+ and GFP- cells on the same y-axis without excessive compression of the less abundant GFP+ cell groups. Imaging and cell quantification were performed while blind to animal identity. 3D reconstructions were performed using 0.5μm z-stacks taken with a 63x oil objective and then rendered with Imaris software (Oxford Instruments). EAAT1 immunolabeling for quantification of knockdown in brain tissue was done using ImageJ to select GFAP+GFP+ area and measure EAAT1 intensity within that area.

#### Pharmacological reagents

Reagents and working dilutions are in the key resources table.

#### Proliferation assays

NSCs were plated 5000 cells per well in a 96 well plate and treated with pharmacological inhibitors or vehicle for 15 minutes prior to the addition of glutamate/aspartate or vehicle. Two days later, 20 μM 5-Ethynyl-2′-deoxyuridine (EdU) was added to the cell culture media for 30 minutes to label NSCs in S-phase. The cells were then fixed in 4% PFA for 20 minutes prior to undergoing a click reaction to detect EdU. Cells were permeabilized for 20 minutes in 0.5% Triton X-100 and incubated in the dark for 30 minutes with copper-dependent click reaction mixture to label EdU directly with fluorophores (Azide-488 or –647). Cells were stored at 4°C in the dark until imaging (see above). After imaging, a standard threshold was applied to the channel containing EdU staining for all images, and the analyze particles function was applied to count the cells with EdU signal above threshold. EdU counts are expressed as relative change to control within experiment to facilitate combining multiple experimental replicates. In generally, a minimum of 3 experimental replicates of each EdU proliferation assay was performed, with multiple well replicates per groups in each individual experiment.

#### Glutamate clearance assay

Glutamate clearance experiments were performed on NSCs grown in 96 well plates to approximately 95% confluency. The growth media was removed and cells were rinsed twice with oxygenated artificial cerebrospinal fluid (aCSF) prepared according to the following recipe (in mM): 116 NaCl, 3.0 KCl, 1.25 NaH2PO4, 23 NaHCO3, 10 glucose, 2.0 MgSO4, and 2.0 CaCl_2_.^71^ Cells were then pre-incubated for 15 minutes with aCSF containing EAAT inhibitors or vehicle before replacing the solution with 50 μL of aCSF containing 5 μM glutamate and EAAT inhibitors or vehicle. Immediately after (T0) and 5, 10, and 20 minutes later, samples (10 μL) were injected and glutamate quantified by uHPLC-ECD.

#### uHPLC-ECD

Glutamate was quantified using uHPLC-ECD (ALEXYS Neurotransmitter Analyzer;Antec Scientific, USA). Samples were pre-column derivatized with o-phthaldialdehyde (OPA) and sulphite. Chromatographic separation was performed via an Acquity UPLC HSS T3 1.0 x 50 mm, 1.8 μm column. The mobile phase A, for separation consisted of 50 mM phosphoric acid, 50 mM citric acid, 0.1 mM EDTA, and 1.5% v/v acetonitrile with a final pH of 3.1. Mobile Phase B, for post-separation flush, consisted of the same mobile phase A but with 50% v/v acetonitrile. The system was operated at a flow rate of 200 μl/min at a pressure of 380 bar. Electrochemical detection used a working potential of 850 mV (vs Ag/AgCl reference) and a range setting of 5 nA/V. Sample peaks were fitted to a glutamate standard curve.

#### Plasmid design and lentiviral packaging

The CRISPRi lentivirus construct was modified from pLV hU6-sgRNA hUbC-dCas9-KRAB-T2a-GFP (Addgene #71237, a gift from Charles Gersbach^29^), which expresses all necessary CRISPRi machinery (both the dCas9-KRAB and sgRNA) from the same plasmid. The UbC promoter was replaced with an EF1α promoter to create pLV hU6-sgRNA EF1α-dCas9-KRAB-T2A-GFP. Additionally, two Esp3I recognition sites were placed after the U6 promoter for restriction cloning of sgRNA insert sequences. The insert sequences were synthesized as single strand oligonucleotides (Integrated DNA Technologies) in the form of 5′ GGACG(N)20 3′ and 5′ AAAC(N′)20C 3′ where (N)20 refers to the sequence of the sgRNA and (N′)20 is the reverse complement. These oligonucleotides were annealed together and ligated with Esp3I-digested pLV hU6-sgRNA EF1α-dCas9-KRAB-T2a-GFP to obtain the final constructs for lentivirus packaging. The sgRNA targeting the region −50 to +300 bp relative to the transcriptional start sequence of Slc1a3 were designed using the CRISPRi function of the online Broad Institute GPP portal.^62^^,^^63^ The top ranked Slc1a3-targeting sgRNA returned by the GPP tool (5′CGGAGTAACAGCTTAGCGAG) and a non-targeting (NT, 5′GCGAGGTATTCGGCTCCGCG) sgRNA^72^ were used to create an EAAT1 KD plasmid and an NT control plasmid. These plasmids were packaged as VSV-G pseudotyped, second-generation lentivirus vectors (titers 1.51 x 10^9^ IFU/mL, EAAT1 KD virus; 1.53 x 10^9^ IFU/mL, NT control virus) by Vigene Biosciences, Inc. These plasmids, including the Esp3I cloning-ready backbone, are available via Addgene (See key resources table).

#### CRISPRi KD *in vitro*

Cultured adult DG NSCs were plated 5000 cells per well in 96 well plates and treated with EAAT1 KD or NT control virus (∼MOI 75–150). Cells were maintained in culture for 8–12 days after virus treatment and were passaged 1–2 times during that timeframe. Cells were treated with 100 μM glutamate or vehicle 2 days before a 30 minute pulse of 20 μM EdU and fixation. Immunofluorescence labeling, EdU detection, and imaging were performed as described above. ImageJ was used to manually draw ROIs around GFP+ and GFP- NSC cell bodies in the brightfield channel and measure the average EAAT1 immunofluorescence signal intensity within the ROI. The number of GFP+EdU+ cells was quantified by manually identifying all GFP+ cells and evaluating colocalization with EdU.

#### Stereotaxic surgery

Mice were anesthetized by isoflurane inhalation (4% induction, 2% maintenance) in oxygen and mounted in the stereotaxic apparatus (Stoelting). The scalp was sterilized with alcohol and betadine before exposing the skull via a single scalpel blade incision. Bilateral bur holes were drilled into the skull, and Hamilton syringes were positioned at A/P −2.0 mm and M/L +2.0 mm before being slowly lowered to a depth of DV –1.9 mm (all coordinates relative to bregma). Mice were administered 0.9 μL EAAT1 KD virus in one hemisphere and 0.9 μL NT control virus in the contralateral hemisphere at a rate of 0.1 μL/min via an automated injector system (Stoelting). The brain hemisphere receiving EAAT1 KD virus was counterbalanced within each timepoint cohort and across sexes. Mice were administered carprofen prior to surgery and daily for 3 days post surgery as well as buprenorphine immediately post surgery. 36 mice total received lentiviral infusion. Cohort 1: 16 (8 male, 8 female) were given 3 injections of EdU (150 mg/kg, i.p.) 4 hours apart 1 week after lentiviral infusion then perfused. GFP expression, indicating proper infusion placement, was confirmed by a blinded observer in 5 NT and 10 EAAT1 KD DGs. Cohort 2: 10 (5 male and 5 female) received a single daily injection of BrdU (150 mg/kg, i.p.; Sigma B5002) on days 6–8 after infusion and 3 injections of EdU on day 21 after lentivirus injection. GFP expression was confirmed by a blinded observer in 8 NT and 8 EAAT1 KD DGs. Cohort 3: 10 mice (5 male and 5 female) received a single daily injection of BrdU (150 mg/kg, i.p.; Sigma B5002) 1 month after infusion, 1 mo before 3 EdU injections and perfusion. GFP expression was confirmed by a blinded observer in 8 NT and 6 EAAT1 KD DGs. All cohorts were perfused two hours after the final EdU injection.

#### Experimental treatment and RNA extraction for RNAseq

NSCs of passage 4 were plated 75,000 cells per well in a 12 well plate and treated with 1 μM TFB-TBOA or vehicle for 15 minutes prior to the addition of 100 μM glutamate or vehicle (n = 3 wells per treatment). After 48 hours, cells were harvested with Accutase and a subset (approximately 75,000 cells) was pelleted prior to RNA extraction using the NucleoSpin RNA Plus XS kit following manufacturer protocol. Assessment of RNA quality with Qubit RNA HS assay kit revealed RIN values of at least 8 for all samples.

#### RNAseq library generation, sequencing, alignment, and transcript quantification

Library generation was performed as previously described^27^ using the Clontech SMART-Seq HT kit. Purified library products were then submitted to HiSeq 4000 paired-end sequencing (Illumina) with a depth of 15–20 million 2 × 150 bp clusters. AdapterRemovalv2.2.0^67^ was used to trim individual FASTQ files for adapter sequences and filter for a minimum quality score of Q20. HISAT2 v2.0.6^68^ was used to exclude reads aligning to a composite reference of rRNA, mtDNA, and PhiX bacteriophage sequences obtained from the NCBI RefSeq. Primary alignment was performed to the mouse reference genome GRCm38p4 using HISAT2. Gene expression values were quantified for all genes described by the GENCODE GeneTransfer Format (GTF) release M14 (mouse) using the featureCounts tool of the Subread package v1.5.1 in stranded mode.^69^^,^^70^ Raw and processed counts are available in GEO accession (see key resources table).

#### Differentially expressed gene (DEG) analysis

DEG analysis was performed using pair-wise comparisons between the following treatment groups: vehicle/vehicle vs glutamate/vehicle, vehicle/vehicle vs vehicle/TFB-TBOA, glutamate/vehicle vs glutamate/TFB-TBOA, and vehicle/TFB-TBOA vs glutamate/TFB-TBOA. For all comparisons, the DESeq R package^64^ was used to compute log_2_fold changes in gene expression between treatment groups, and genes with a Benjamini-Hochberg adjusted p value <0.05 were considered as DEGs.

#### Gene ontology enrichment (GO) analysis and clustering

GO analysis of DEGs was performed using the ClueGo plugin v.2.5.7^66^ for Cytoscape v3.9.0.^65^ Upregulated and downregulated DEGs were analyzed separately. DEGs were compared to the GO term annotations within the reference ontology GO_BiologicalProcess-EBI-UniProt-GOA-ACAP-ARAP_08.05.2020_00h00, and a two-sided hypergeometric test was performed with a Bonferroni adjusted p value < 0.05 considered as significant enrichment. Similar GO terms were clustered using iterative kappa score grouping.

#### Diagrams and graphical abstract

Diagrams and graphical abstract were created using Biorender.com software.

### Quantification and statistical analysis

Statistical analysis was performed using GraphPad Prism version 9 or higher, except for RNAseq data, which is described above. Generally, if experiments used a 2x2 factor design, 2-way ANOVA was used with error-corrected posthoc tests. If experiments used a 3x2 factor design, 3-way ANOVA was used with error-corrected posthoc tests. If experiments only had 2 groups, non-parametric (Mann Whitney, for log-transformed data) or parametric comparisons (t-test for non-transformed data) were used. p < 0.05 was defined as significant. Details of statistical tests are in Table S4, and details of sample size are in figure legends.

## Data Availability

• RNA-seq data have been deposited at GEO and are publicly available as of the date of publication. Accession numbers are listed in the key resources table. Microscopy data and qPCR data reported in this paper will be shared by the lead contact upon request. • This paper does not report original code. • Any additional information required to reanalyze the data reported in this paper is available from the lead contact upon request.

## Supplemental information

Document S1. Figures S1–S6

Table S1. Glutamate and EAAT1KD RNA-seq DEGs, supplement to Figure 6

Table S2. RNA-seq GO terms derived from DEGs, supplement to Figure 6

Table S3. RNA-seq raw normalized counts, supplement to Figure 6

Table S4. Statistical tests, supplement to STAR Methods
