# Supplementary material for: Excitatory amino acid transporter 1 supports adult hippocampal neural stem cell self-renewal
Source: iScience. 2023 Jun 8;26(7):107068. doi: 10.1016/j.isci.2023.107068 (PMC10391730; doi:10.1016/j.isci.2023.107068)
Supplement: Document S1. Figures S1–S6 [file mmc1.pdf]

iScience, Volume *26*

Supplemental information

Excitatory amino acid transporter 1 supports adult hippocampal neural stem cell self-renewal

Joshua D. Rieskamp, Ileanexis Rosado-Burgos, Jacob E. Christofi, Eliza Ansar, Dalia Einstein, Ashley E. Walters, Valentina Valentini, John P. Bruno, and Elizabeth D. Kirby

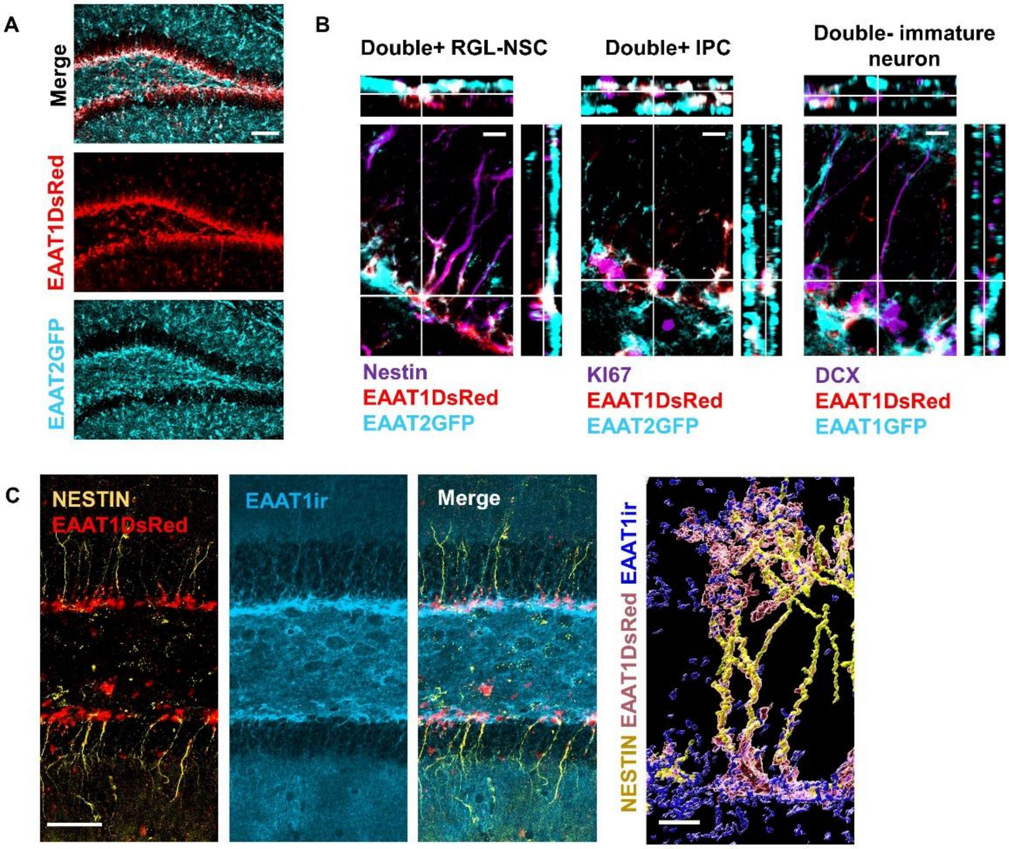

Figure S1: Adult hippocampal NSCs express EAATs, Supplement to Fig 1

A) EAAT1DsRed+EAAT2GFP+ fluorescence in the adult DG. Scale = 100 µm.

B) Orthogonal projections of example: EAAT1DsRed+EAAT2GFP+ (double+) Nestin+ radial glia like (RGL) NSC, EAAT1DsRed+EAAT2GFP+ (double+) Ki67+ IPC, EAAT1DsRed-EAAT2GFP- (double-) DCX+ immature neuron. Scale = 10 µm.

C) Nestin immunolabeling in DG of EAAT1DsRed+ co-localized with EAAT1 immunoreactivity (ir). Left is merged z-stack. Right is 3D reconstruction showing an example Nestin+EAAT1DsRed+ radial glia like NSC with EAAT1ir in the cell body and apical terminals.

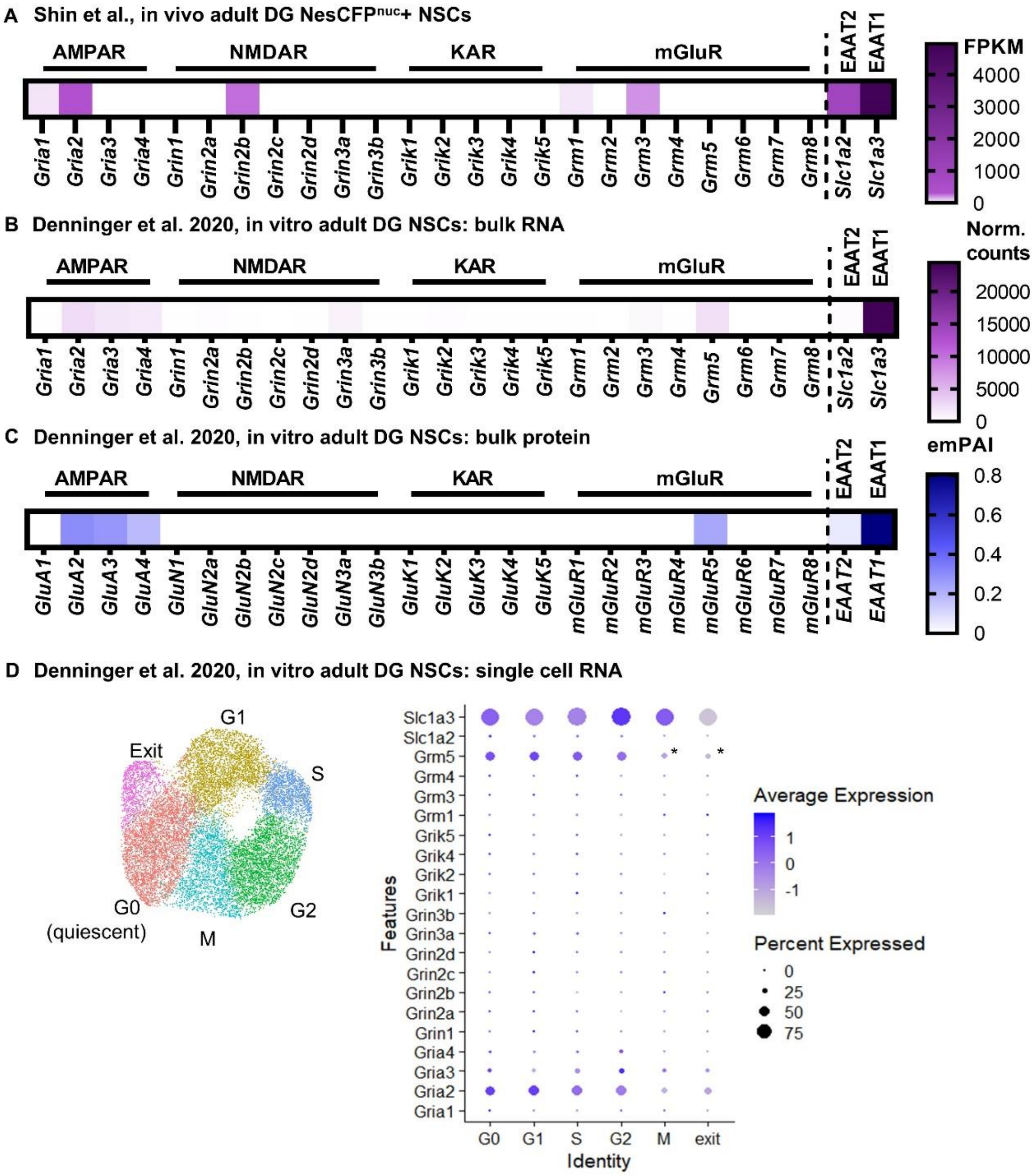

Figure S2: In vitro DG-derived NSCs express high levels of EAAT1 and low levels of glutamate receptors throughout the cell cycle, Supplement to Fig 2

A) FPKM of glutamate receptor and EAAT1/2 genes from Shin et al., 2015.

B) Transcript length-normalized RNAseq counts of glutamate receptor genes and EAAT1/2 from cultured adult DG NSCs in Denninger et al., 2020.

C) emPAI length-normalized semi-quantitiative spectral counts for glutamate receptor and transporter proteins from cultured adult DG NSCs in Denninger et al., 2020.

D) Single cell RNAseq of cultured DG NSCs from Denninger et al., 2020 reflects a mixture of active and quiescent NSCs plus a cycle-exiting population, as shown in UMAP. Dot plot shows average

normalized RNA count per cell (color scale) and percent of cells expressing (dot size) glutamate transporter and receptor genes. * = differentially expressed gene for cluster in original analysis.

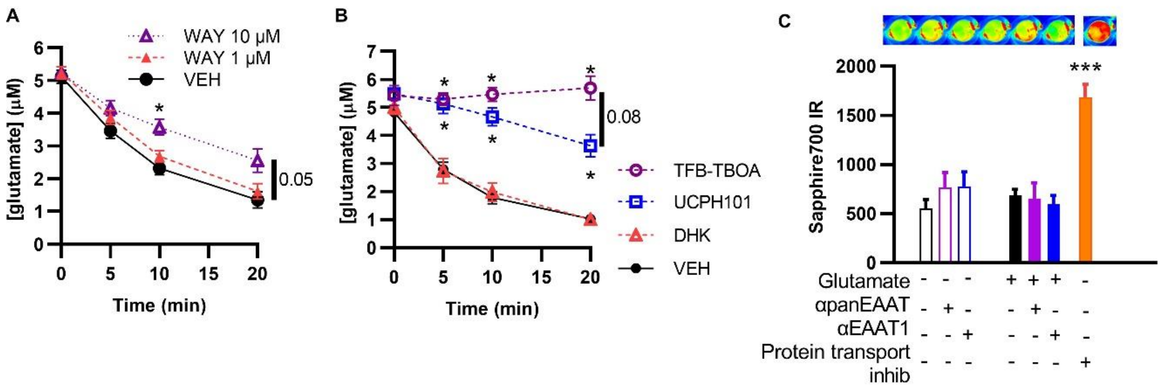

Figure S3: EAAT1 glutamate transport is highly active in in vitro NSCs, and its inhibition does not increase cell death, Supplement to Fig 3

A) Glutamate in NSC conditioned media as measured by uHPLC after a 5 µM pulse after pre-treatment with an EAAT2-selective (1 µM) and an EAAT1and2 selective (10 µM) dose of WAY213613. *p<0.05 vs 1 µM and vs 10 µM. Mean ± SEM of N = 6 independent experiments.

B) Glutamate in NSC conditioned media as measured by uHPLC after a 5 µM pulse after pre-treatment with a panEAAT inhibitor (TFB-TBOA), an EAAT1 inhibitor (UCPH101), or and EAAT2 inhibitor (DHK). *p<0.05 Tukey's comparisons within timepoint. Mean ± SEM of N = 3 independent experiments.

C) Sapphire IR signal in cultured NSCs after treatment with glutamate +/- panEAAT inhibitor (TFB-TBOA) or EAAT1 inhibitor (UCPH101). Protein transport inhibitor at toxic dose is positive control for presence of apoptosis in Sapphire IR700 assay. ***p<0.001 Tukey's multiple comparisons. Mean ± SEM of N = 3-6 replicates/experiment, 2 independent experiments. Top shows representative image of Sapphire IR signal in treatment groups.

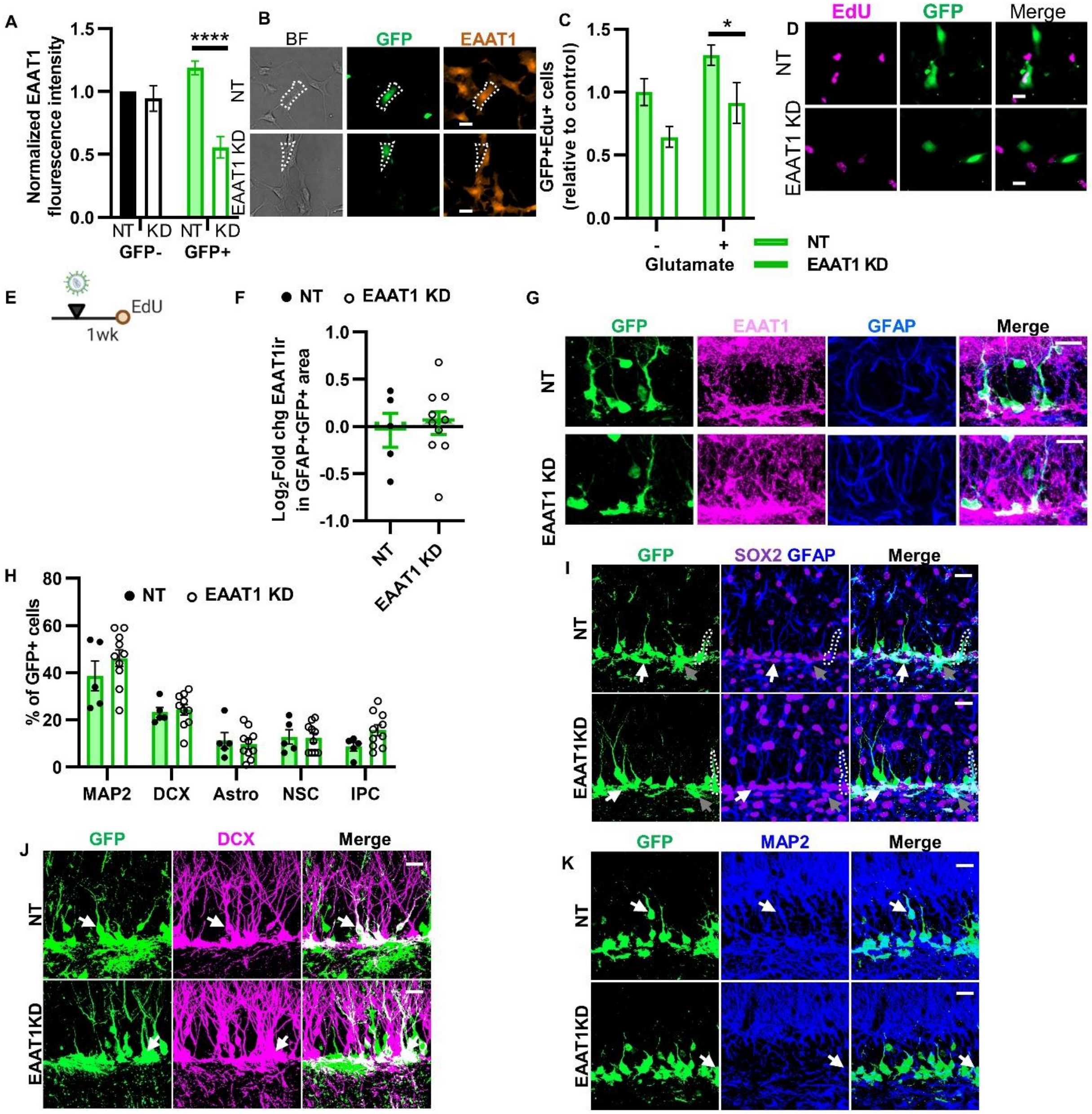

Figure S4: EAAT1 expression after CRISPRi knockdown and cell phenotypes 1 week after CRISPRi infusion, Supplement to Figure 4

A) EAAT1 immunoreactive intensity in cultured NSCs infected with NT and EAAT1 KD CRISPRi lentiviral vectors. ****p<0.0001 Sidak's multiple comparisons within GFP type. Mean ± SEM of N = 4 independent infections.

B) Representative images of EAAT1 labeling coupled with GFP and brightfield to visualize all NSCs. Scale = 10 µm.

C) EdU+GFP+ cell counts in cultured NSCs infected with NT and EAAT1 KD CRISPRi lentiviral vectors. Mean ± SEM of N = 3 replicates/infection, 3 independent infections.

D) Representative images of EdU labeling coupled with GFP in NSCs infected with NT and EAAT1 KD CRISPRi lentiviral vectors. Scale = 10 µm.

E) Timeline of mice infused with NT or EAAT1 KD CRISPRi lentiviral vectors in the DG then perfused 1 week later.
F) EAAT1 immunolabeling intensity within GFP+GFAP+ area. Mean ± SEM of N = 5-8 individual mice (points shown).
G) Representative image of EAAT1 labeling coupled with GFP and GFAP at 1 week after CRISPRi infusion. Co-labeling becomes white in merged image. Scale = 20 µm.
H) Percent of cell types expressing GFP at 1 week after CRISPRi infusion. Cell types identified by MAP2, DCX, SOX2 and GFAP expression.
I) Representative images of SOX2, GFAP, and GFP immunolabeling at 1 week after CRISPRi infusion. White arrows point to IPCs, grey arrows point to Astrocytes, SOX2+GFAP+ NSC outlined in white. Scale = 20 µm.
J) Representative images of DCX and GFP co-labeling at 1 week after CRISPRi infusion. White arrows point to GFP+DCX+ cells. Scale = 20 µm.
K) Representative images of MAP2 and GFP co-labeling at 1 week after CRISPRi infusion. White arrows point to GFP+MAP2+ cells. Scale = 20 µm.

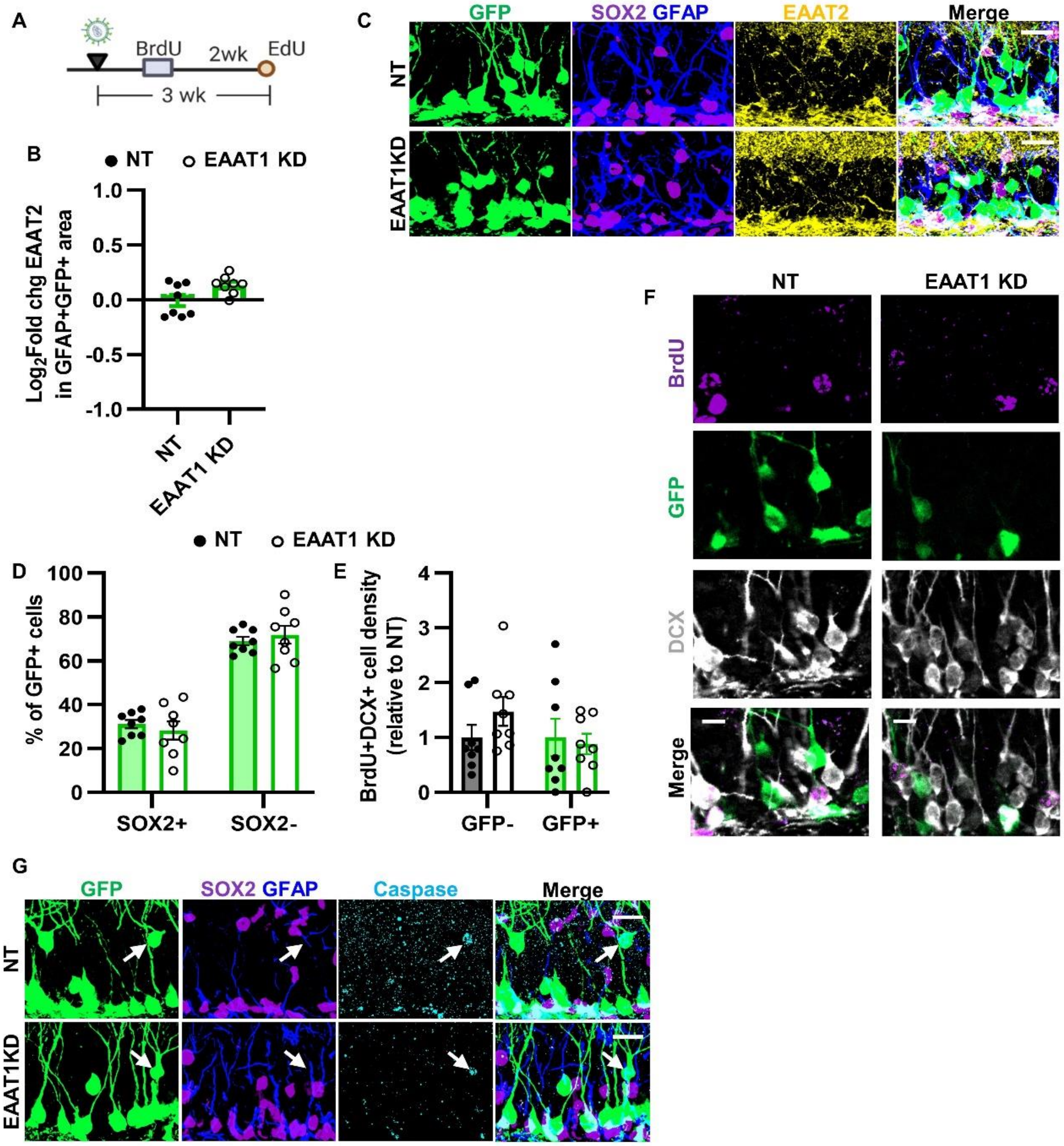

Figure S5: EAAT2 protein levels, aCasp3 labeling and cell phenotypes 3 weeks after CRISPRi infusion, Supplement to Fig 4, 3 week data

A) Timeline of mice infused with NT or EAAT1 KD CRISPRi lentiviral vectors in the DG then perfused 3 week later.

B) EAAT2 immunolabeling intensity within GFP+GFAP+ area of EAAT1KD or NT mice 3 weeks after CRISPRi infusion. Mean ± SEM of N = 8 individual mice (points shown).

C) Representative image of EAAT2, GFP, SOX2 and GFAP immunolabeling at 3 weeks after CRISPRi infusion. Co-labeling becomes white in merged image. Scale = 20 µm.

D) Percent of GFP+ cells co-expressing SOX2 3 weeks after CRISPRi infusion.

E) Density of GFP+ and GFP- BrdU+DCX+ immature neurons/neuroblasts 3 weeks after CRISPRi infusion.

F) Representative image of BrdU, GFP, DCX co-labeling 3 weeks after CRISPRi vector infusion. Scale = 10 µm.

G) Representative images of clCas3, GFP, SOX2, and GFAP immunolabeling at 3 week after CRISPRi infusion. White arrows point out clCas3+ cells. Scale = 20 µm. See quantification in Fig. 4.

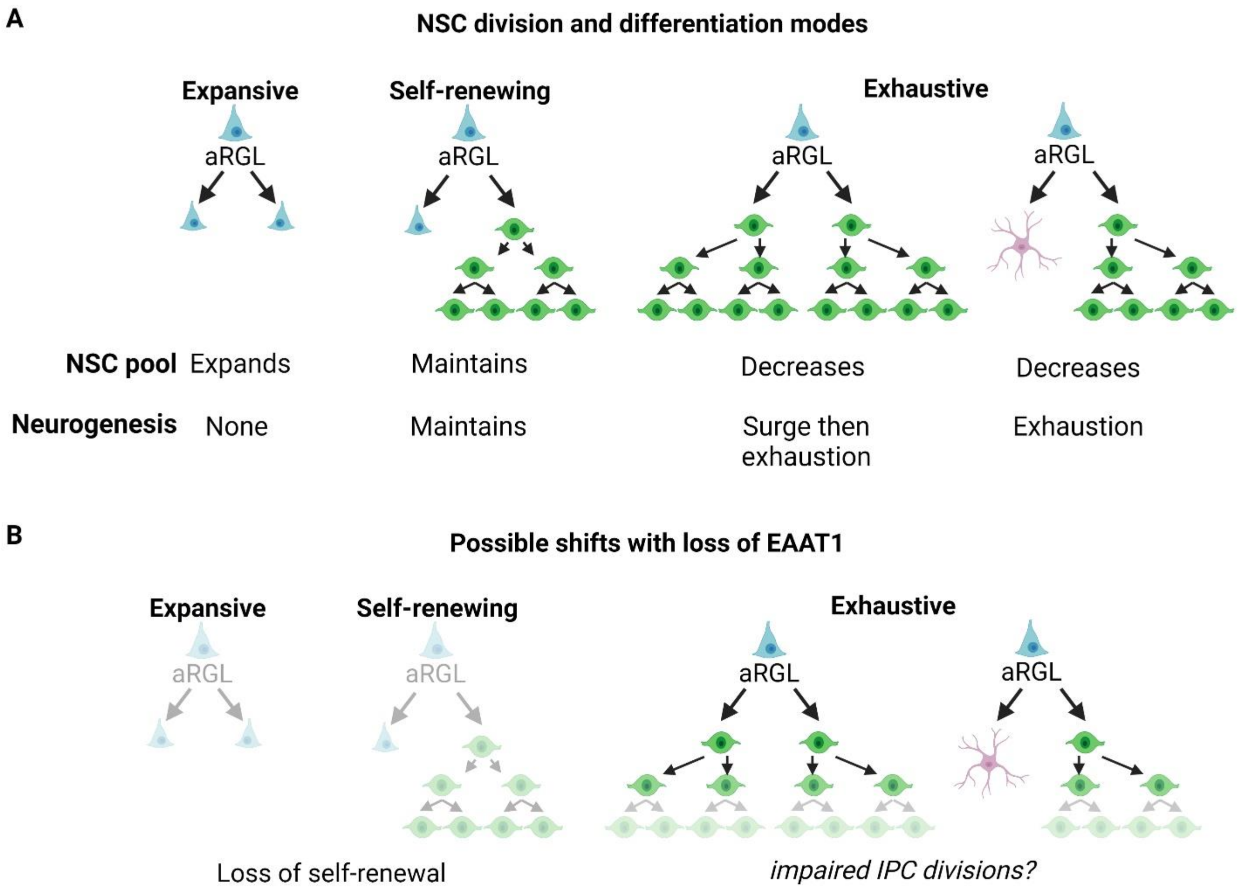

Figure S6: Proposed model for how NSC proliferation and differentiation modes are altered with loss of EAAT1, Supplement to Discussion

A) Diagram of NSC proliferation manners with EAAT1-mediated glutamate transport, showing that NSCs have varied manners of proliferation. Active NSCs proliferate in a mostly self-renewing manner, with some expansive and exhaustive proliferations as well, balancing NSC maintenance. Below are outcomes for the NSC pool and NSC-derived neurons for each of these proliferation manners.

B) Diagram of possible alterations to NSC proliferation manner without EAAT1-mediated glutamate transport. Loss of self-renewal could derive from loss of expansive and/or self-renewing divisions. The net effect of either of those changes would be a shifted balance towards more exhaustive proliferation, leading to loss of NSC pool.
